# Overcoming the Hurdles of Autologous T-Cell-Based Therapies in B-Cell Non-Hodgkin Lymphoma

**DOI:** 10.3390/cancers12123837

**Published:** 2020-12-18

**Authors:** Jaco A. C. van Bruggen, Anne W. J. Martens, Sanne H. Tonino, Arnon P. Kater

**Affiliations:** 1 Department of Hematology, Amsterdam UMC, University of Amsterdam, 1105 AZ Amsterdam, The Netherlands; j.a.vanbruggen@amsterdamumc.nl (J.A.C.v.B.); a.w.martens@amsterdamumc.nl (A.W.J.M.); s.h.tonino@amsterdamumc.nl (S.H.T.); 2 Department of Experimental Immunology, Amsterdam UMC, University of Amsterdam, 1105 AZ Amsterdam, The Netherlands; 3 Cancer Center Amsterdam, 1105 AZ Amsterdam, The Netherlands; 4 Amsterdam Infection & Immunity Institute, 1105 AZ Amsterdam, The Netherlands; 5 Lymphoma and Myeloma Center Amsterdam, LYMMCARE, 1105 AZ Amsterdam, The Netherlands

**Keywords:** immunotherapy, B-NHL, CLL, CAR T-cells, immune checkpoint blockade, bispecific antibodies, T-cell dysfunction

## Abstract

**Simple Summary:**

The activity of novel therapies that utilize patient’s own T-cells to induce remission of B-cell non-Hodgkin lymphoma (B-NHL), including chronic lymphocytic leukemia (CLL), is still suboptimal. In this review, we summarize the clinical efficacy of T-cell-based therapies in B-NHL and provide a biologic rationale for the observed (lack of) responses. We describe and compare the acquired T-cell dysfunctions that occur in the different subtypes of B-NHL. Furthermore, we discuss new insights that could enhance the efficacy of T-cell-based therapies for B-NHL and CLL.

**Abstract:**

The next frontier towards a cure for B-cell non-Hodgkin lymphomas (B-NHL) is autologous cellular immunotherapy such as immune checkpoint blockade (ICB), bispecific antibodies (BsAbs) and chimeric antigen receptor (CAR) T-cells. While highly successful in various solid malignancies and in aggressive B-cell leukemia, this clinical success is often not matched in B-NHL. T-cell subset skewing, exhaustion, expansion of regulatory T-cell subsets, or other yet to be defined mechanisms may underlie the lack of efficacy of these treatment modalities. In this review, a systematic overview of results from clinical trials is given and is accompanied by reported data on T-cell dysfunction. From these results, we distill the underlying pathways that might be responsible for the observed differences in clinical responses towards autologous T-cell-based cellular immunotherapy modalities between diffuse large B-cell lymphoma (DLBCL), chronic lymphocytic leukemia (CLL), follicular lymphoma (FL), mantle cell lymphoma (MCL), and marginal zone lymphoma (MZL). By integration of the clinical and biological findings, we postulate strategies that might enhance the efficacy of autologous-based cellular immunotherapy for the treatment of B-NHL.

## 1. Introduction

B-cell non-Hodgkin lymphomas (B-NHL) arise during different stages of B-cell development, which is reflected by differences in biological and clinical characteristics. This also explains their distinct sensitivity to therapeutic modalities. Current treatment of B-NHL includes DNA crosslinking agents such as cyclophosphamide, bendamustine, and purine analogs such as fludarabine in combination with CD20 targeting antibodies. In more aggressive tumor types, inhibitors of DNA synthesis such as doxorubicin are added. Our increased understanding of oncogenesis and the relevance of crosstalk between immune cells and cancer cells in the tumor microenvironment (TME) has led to the development of targeted therapies in B-NHL, such as inhibitors of the B-cell receptor signaling pathway, and inhibitors of apoptosis regulating proteins such as the Bcl-2 binding drug venetoclax [1,2]. Furthermore, the development of antibody drug conjugates, such as polatuzumab vedotin (targeting CD79b) or brentuximab vedotin (targeting CD30), has resulted in an increased armamentarium of effective drugs with acceptable toxicity [3,4,5]. These agents are highly active either as a single agent or in combinations, but thus far are not curative, and resistance towards these agents will ultimately develop [6,7]. The next therapeutic frontier in B-NHL is autologous T-cell-based therapy, which includes immune checkpoint blockade (ICB), bispecific antibodies (BsAbs), and chimeric antigen receptor (CAR) T-cell therapy.

ICB reverses inhibitory interactions between the T-cell and cancer cell, thereby improving cytotoxicity of tumor recognizing T-cells and inducing cancer cell lysis. Within the tumor microenvironment, chronic stimulation of tumor infiltrating lymphocytes (TILs) leads to T-cell exhaustion, which hampers proper anti-tumor responses. Blocking of inhibitory receptors, such as programmed death-1 (PD-1) or cytotoxic T-lymphocyte-associated antigen 4 (CTLA-4), on T-cells, or their ligands on tumor or adjacent T-cells, potentiates anti-tumor responses. Investigations of these antibodies in hematological malignancies were prompted by the success of ICB in solid tumors such as melanoma and non-small cell lung cancer [8,9] and led to impressive results in Hodgkin lymphoma (HL) [10].

In contrast to ICB, BsAbs induce tumor cell killing without the need for specific tumor cell recognition by autologous T-cells. BsAbs tether T-cells and cancer cells via CD3 on T-cells and a tumor-associated target antigen on cancer cells resulting in direct tumor cell killing. Blinatumumab was the first Food and Drug Administration (FDA)-approved CD3xCD19 bispecific T-cell engager (BiTE). This BsAb is currently only approved for relapsed/refractory acute lymphoblastic leukemia (ALL), in which trials have shown complete remission (CR) rates of around 33% [11,12].

Similar to BsAbs, CAR T-cells act through direct recognition of cancer cells. T-cells acquire CARs through retroviral or lentiviral vectors, which requires ex vivo transduction, activation, and expansion. First generation CARs consisted of an antigen binding moiety (ScFv) covalently linked to CD3 zeta, allowing tumor antigen recognition and subsequent T-cell activation [13,14]. Second generation CARs contain an additional (costimulatory) CD28 or CD137 (4-1BB) intracellular domain, which greatly enhances CAR T-cell function [15,16,17]. High efficacy of CAR T-cell therapy was seen in clinical trials in ALL, in which 90% of the patients achieved CR [18,19]. Since then, CAR T-cell therapy has been translated to other types of hematologic malignancies including B-NHL.

Responses to aforementioned immune therapies are still suboptimal in B-NHL, including chronic lymphocytic leukemia (CLL). By exploring clinical data and connecting them to ex vivo and in vitro observations on T-cell (dys)function, we propose underlying mechanisms of treatment failure for the most common types of B-NHL, including diffuse large B-cell lymphoma (DLBCL), chronic lymphocytic leukemia (CLL), follicular lymphoma (FL), mantle cell lymphoma (MCL), and marginal zone lymphoma (MZL). These data are integrated in order to suggest possible solutions to the current hurdles of autologous-based T-cell therapies in B-NHL.

## 2. Clinical Data

### 2.1. Immune Checkpoint Blockade

Although response rates were modest in early trials, patients that responded to ICB were reported to be in remission for over a year [20,21,22]. This prompted further investigation of ICB in B-NHL (Table 1). Studies using ipilimumab (anti-CTLA-4) or pembrolizumab (anti-PD-1) showed overall response rates (ORR) between 0–11% in B-NHL [23,24,25]. Whereas CLL patients showed no response upon treatment with pembrolizumab, four of 9 (44%) CLL, patients with Richter’s transformation (RT) had a response [25]. Initially, nivolumab (anti-PD-1) appeared to be more promising with an ORR of 36% and 40% for DLBCL and FL, respectively [26]. However, these initial high responses could not be confirmed in a large follow-up trial, where DLBCL patients were subdivided based on their ineligibility for autologous hematopoietic stem cell therapy (auto-HSCT) or having relapsed after auto-HSCT [27]. The 87 patients included in the auto-HSCT failed group had an ORR of 10%, while the ORR in the auto-HSCT ineligible group (*n* = 34) was only 3% [27]. Primary mediastinal large B-cell lymphoma (PMBCL) does respond exceptionally well to ICB compared to the others B-NHL subtypes with an ORR of 48% (CR 33%) and a median duration of response (DOR) was not reached at a median follow-up time of 29 months [28].

These trials indicate that ICB monotherapy is not sufficient to elicit significant responses in B-NHL. Therefore, research has focused on combining ICB antibodies or adding other treatment modalities to enhance efficacy. Unfortunately, combining ipilimumab and nivolumab in B-NHL patients, FL or DLBCL, resulted in a modest partial response (PR) of three out of 15 patients (ORR 20%) [29]. In contrast, the combination of pembrolizumab or pidilizumab (anti-PD-1) with rituximab greatly improved clinical outcomes in relapsed FL compared to ICB monotherapy (ORRs between 64 and 66%) [30,31]. It remains to be elucidated whether ICB significantly improves the activity of rituximab-based therapy, since single agent rituximab for FL results in ORRs between 40% and 72% [32,33,34]. The combination of the anti-CD20 monoclonal obinutuzumab with atezolizumab (anti- programmed death-ligand 1, PD-L1) resulted in an ORR of 57% in 26 FL patients [35]. Addition of bendamustine to a combination of obinutuzumab and atezolizumab further increased the response rate in previously untreated FL. Twelve out of 15 patients showed a response (ORR 80%), of which 10 patients had CR [36]. Even though in FL the combination of obinutuzumab and atezolizumab seems promising, in 23 DLBCL patients the ORR was only 16% [35]. However, the combination of atezolizumab with rituximab, cyclophosphamide, doxorubicin, vincristine, and prednisone (R-CHOP) in untreated DLBCL led to responses in 35 out of 40 patients (ORR 88%), with 31 obtaining CR, and therefore might be favorable compared to R-CHOP alone [37].

Combinations of ICB and lenalidomide have also been studied. Preliminary data from a phase I trial combining nivolumab with lenalidomide in 10 B-cell lymphoma patients (including DLBCL, HL, high-grade B-cell lymphoma and lymphoplasmacytic lymphoma) showed an ORR of 30% [38]. Another study tested durvalumab (anti-PD-L1) as monotherapy, or with addition of lenalidomide with or without rituximab [39]. The arm investigating durvalumab and lenalidomide was prematurely closed due to (expected) toxicity of this combination [39]. In this arm, four out of five (80%) of FL patients and two out of seven (29%) DLBCL patients showed a response upon treatment with ICB and lenalidomide, while durvalumab monotherapy did not lead to responses in both malignancies [39]. Ipilimumab and lenalidomide were also administered to a small number of patients with high-risk DLBCL, CLL, FL or MCL [40]. In seven patients that were stratified as high-risk disease after autologous stem cell transplantation (SCT), both ORR and CR rate were 86%, while the ORR was 70% in the 10 patients that relapsed following allogeneic SCT, with a CR rate of 40% [40]. These data suggest a possible benefit of lenalidomide and ipilimumab in the post-SCT setting.

The combination of nivolumab with the Bruton’s tyrosine kinase (BTK) inhibitor ibrutinib was tested in a large phase I/II study. ORR rates in CLL/small lymphocytic leukemia (SLL) (36 patients), CLL with RT (20 patients), FL (40 patients) and DLBCL (45 patients) were 61%, 65%, 33% and 36%, respectively [41]. Except for the CLL with RT group, the ORR of the other groups were comparable to ibrutinib monotherapy [26,42,43,44]. These results were similar to a more recent trial in which ibrutinib was combined with durvalumab, which yielded responses in 15 out of 59 patients with FL or DLBCL (ORR 25%) [45].

Again, relatively high response rates are seen in PBMCL patients treated with a combination of ICB (nivolumab) with the anti-CD30 antibody-drug conjugate brentuximab vedotin (ORR of 73%) with CR in 11 patients (35%) [46].

Together, these results indicate that, unlike in HL, results for ICB in B-NHL are thus far disappointing, except for PMBCL. Although combinations, such as with rituximab or lenalidomide, seem to increase response rates, it remains to be elucidated whether a true synergistic, or at least additive, effect exists.

### 2.2. Bispecific Antibody Therapy

Multiple studies have investigated BsAbs in B-NHL (Table 2). The first phase I study of blinatumomab in relapsed/refractory B-NHL showed an overall response rate (ORR) of 80% for FL (15 patients), 71% for MCL (seven patients) and 55% for DLBCL (11 patients), with a median DOR of 404 days [48]. Similar results were reported in a phase II study for relapsed/refractory DLBCL using blinatumomab [49]. Of the 21 patients that received the target dose, 9 obtained a response (ORR 43%), of which four had CR, with a median DOR of 11.6 months and progression free survival (PFS) of 3.7 months (median follow-up of 15.0 months) [49]. In line with this, Coyle et al. showed an ORR of 37% and CR of 22% in relapsed/refractory B-NHL, of which the majority of patients were diagnosed with DLBCL (*n* = 34 of 41 included patients) [50]. Although these first results with blinatumomab were encouraging, albeit most responses were partial, preliminary data show that response rates can be increased by using a combination of blinatumomab and lenalidomide [51]. This combination resulted in an ORR of 83% with 50% CR in 18 relapsed/refractory B-NHL patients including DLBCL (seven patients), MCL (three patients), FL (three patients) and MZL (one patient) [51]. Blinatumomab has also been tested as a consolidation treatment in DLBCL. Patients received first-line treatment consisting of six cycles of rituximab and chemotherapy followed by at least one or two cycles of blinatumomab. Of 28 evaluable patients, 25 had a response (ORR 89%) after blinatumomab treatment, and with a median follow-up time of 8.6 months, 93% of patients were still alive [52].

Treatment with mosunetuzumab, a CD3xCD20 BsAb, resulted in an ORR of 64% and CR of 42% in 64 patients with indolent B-NHL, mainly consisting of FL patients [53]. In contrast, the ORR and CR rate were significantly lower (34% and 18%, respectively) in 119 patients with more aggressive B-NHL (DLBCL or transformed FL) [53]. Surprisingly, patients who had relapsed or were refractory to CAR T-cell therapy could mount effective anti-lymphoma responses upon treatment with mosunetuzumab, with an ORR of 44% [53]. Preliminary results with epcoritamab, a CD3xCD20 bispecific that is administered subcutaneously, showed promising findings in patients with DLBCL and FL. Although cohorts are still small, five out of nine patients with high grade B-cell lymphoma that received dosages ≥6 mg had a response (ORR 56%), of whom four had CR [54]. In a small cohort of six FL patients that received ≥0.76 mg, all obtained a PR (ORR 100%) [54]. Another trial using CD3xCD20 bispecific antibody REGN1979 showed that B-NHL patients who had previously received an anti-CD20 antibody had an ORR of 26% with 80 days as median DOR [55]. In this cohort, objective responses were reported in patients with DLBCL (22 patients), FL (11 patients), MCL (two patients) and MZL (two patients) [55]. A recent update of this study showed promising data for FL and DLBCL. Administration of ≥5 mg of REGN1979 led to responses in 26 of 28 FL patients (ORR 93%), with CR in 21 patients (75%) [56]. In DLBCL patients that had not received CAR T-cell therapy, responses were mainly observed in increased REGN1979 dosages of ≥ 80 mg; six out of 10 patients showed a response, which were all CRs. However, after the failure of CAR T-cell therapy, responses were only observed in seven out of 21 patients (ORR 33%), of which five were CR [56]. Additionally, combinations of a CD3xCD20 antibody and an anti-CD20 antibody seem feasible. In a phase I trial, the CD3xCD20 BsAb glofitamab was given concurrently with obinutuzumab. Patients that were included had aggressive B-NHL or FL. In the 21 included patients, the ORR and CR was 48% and 43%, respectively; the 10 patients that received the highest dosage of glofitamab showed remarkable high responses: an ORR of 90% with a CR rate of 80% [57].

In general, these studies show that reasonable responses against B-NHL can be elicited by BsAb monotherapy. Although patients with indolent lymphomas such as MCL and MZL are currently underrepresented, enhanced efficacy of BsAbs towards indolent lymphomas compared to DLBCL is observed (Table 2). For CLL, no clinical data are available, although in vitro studies seem promising [58,59,60,61,62]. In FL and DLBCL, combinations of BsABs with chemotherapy, lenalidomide and/or CD20 antibodies are favorable, although final results of these trials have to be awaited [51,52].

### 2.3. Chimeric Antigen Receptor Therapy

Virtually all CAR construct studies in trials thus far are directed against the pan B-cell marker CD19 and contain either the intracellular domain of the co-stimulatory receptor CD28 (28z) or 4-1BB (BBz). Since 28z and BBz CARs are functionally different, we will discuss them separately (Table 3). Currently all CAR trials use a one-time infusion strategy. As only CRs are associated with long-term responses, we will focus on CR rates [63,64,65]. The first 28z CAR T-cell trials in B-NHL demonstrated varying CR rates of 0–25% [66,67,68,69]. The minority of patients that did reach CR achieved long-lasting remissions, with DORs ranging from 8 weeks to 15 months [66,67,68,69]. Since then, CAR T-cell therapy for B-NHL has been further optimized as demonstrated by improved CRs ranging from 22% to 62% in subsequent trials [70,71,72,73]. The DOR in these studies ranged from 6 weeks to 52.8 months [70,71,72,73]. Large scale clinical trials recruiting patients with specific types of B-NHL allowed better determination of the efficacy per tumor type. In DLBCL patients (*n* = 108) treated with 28z CAR, a CR rate of 58% was observed. The DOR was 11 months (PFS of 5–9 months) [74]. Similarly, an interim analysis from the ZUMA-5 trial demonstrated a CR rate of 79% in 80 FL and 14 MZL patients, and ongoing responses were observed in 68% of the patients [75]. In another study, 60 evaluable MCL patients demonstrated at least a CR rate of 67% after 7 months of follow-up. The median time to initial response was 1 month, and at median follow-up of 12.3 months, 57% of the 60 evaluable patients were still in remission [65].

Early and small trials (that recruited ≤ 50 patients) using BBz CAR T-cells yielded CRs of 19.5–87.5% [76,77,78,79,80]. Lasting DORs were obtained and ranged between 7.7–39.9 months [76,77,78,79,80]. A large-scale study demonstrated efficacy of BBz CAR T-cells in 93 DLBCL patients, of which 40% had CR. Twelve months after the initial response, the relapse free survival was 65% amongst all patients, and 79% among patients with CR [64]. Another large study recruited 342 DLBCL patients, of which 255 patients were evaluable for response. The CR was 53% with a median DOR of 13.3 months. Patients had a PFS of 6.8 months with a median OS of 19.9 months [81].

To our knowledge, only one study has directly compared the clinical efficacy of 28z and BBz CARs. Three patients received 28z CARs and six patients received BBz CARs. Unfortunately, the 28z CAR was not well tolerated. Eight patients were evaluable for response, of which one had MZL (received BBz CAR), three DLBCL (two BBz and one 28z), and five FL (three BBz and two 28z). Eighty-seven percent of the patients had CR with an ORR of 87% after 3 months [82].

In an effort to search for better responses to CAR T-cell therapy, multiple studies have investigated combinations of CAR with ibrutinib or anti-PD-1. For example, 22 patients with CLL who had received ibrutinib as a prior therapy received BBz CARs, which resulted in CR in 45.5% [83]. Improved responses may be the result of enhanced T-cell engraftment and CAR T-cell generation, both of which correlated with clinical response [84]. Cao et al. treated 11 patients with BBz CARs and nivolumab, of which 10 patients had DLBCL and one had Burkitt lymphoma. The CR was 45.5% with a median PFS of 6 months [85].

Based on the clinical results described above, it becomes clear that CAR T-cell therapy can yield impressive and lasting responses in B-NHL patients. Especially in DLBCL, FL, MCL, and MZL, CAR T-cell therapy yields high CR rates but still falls well below of what is observed in ALL, with the exception of very few clinical trials (Table 3). The most striking observation is that the lowest CRs are observed in patients with CLL in both 28z and BBz CAR T cell studies. Perhaps the low efficacy of CAR T cell therapy in CLL can be explained by the coinciding immune dysfunction that is observed in CLL patients. In the next section, we will discuss this in-depth for each type of B-NHL discussed before.

## 3. T-Cell Immune Surveillance in NHL

From the clinical observations, it follows that efficacy of autologous T-cell-based cellular therapies differs between lymphoma subtypes and between treatment modalities, with the lowest responses for ICB, and that in general, such treatments are less effective in the discussed lymphoma entities than in HL (ICB) and ALL (BsABs and CAR T-cells). In recent years, it has become apparent that patients with B-NHL acquire alterations in T-cell differentiation and function. These T-cell abnormalities affect responses to cellular therapies but are not similar among the different lymphoma subtypes. In this section, we predominantly focus on changes in phenotype and function of T-cells in B-NHL.

### 3.1. Mutational Load in B-NHL

Successful ICB depends on recognition of tumor cells by immune effector cells such as T-cells. These T-cells often recognize antigens that arise due to mutations within tumor cells. Indeed, a clear correlation exists between tumor mutational load and efficacy of ICB [89,90]. Although tumor-specific T-cells have been found in a subset of CLL and MCL patients [91,92,93], the mutational load of B-cell lymphomas is remarkably lower than tumors known to be sensitive to ICB treatment [89]. These results imply that only a subset of lymphomas with high tumor mutational burden might be sensitive to ICB.

### 3.2. T-Cell Skewing

T-cells can be subdivided into naive (CD45RA^+^/CD27^+^/CCR7^+^), memory (including central memory (CD45RA^−^/CD27^+^/CCR7^+^)), effector memory (CD45RA^−^/CD27^−^/CCR7^−^) T-cells, and terminally differentiated effector T-cells (CD45RA^+^/CD27^−^/CCR7^−^) [94,95]. Of these subsets, it is the memory T-cell subset that persists in vivo and has high proliferative capacity, while effector cells have superior effector function [96]. Skewing of these T-cell subsets has mainly been studied in CLL, FL and MZL, although research has focused on different compartments within these lymphoma types, whereas observations of T-cell skewing in CLL are mostly derived from analyzing peripheral blood (PB), for other lymphomas, lymph node-(LN)-derived T-cells have been the main focus.

In PB of CLL, absolute CD4^+^ and CD8^+^ numbers are increased [97,98,99,100], and CD4:CD8 ratios are inverted upon disease progression [101,102]. In addition, CD4^+^ and CD8^+^ effector memory T-cells are expanded at the cost of naive T-cells [98,101,102,103]. Compared to PB, in LN of CLL patients, an increased amount of central memory T-cells were observed [104]. Both T helper (Th)1 and Th2 cells are increased in absolute numbers [103] but data on skewing of Th1/Th2 balance are conflicting. Skewing towards a more pro-tumoral Th2 phenotype in which CD4^+^ T-cells produce interleukin (IL) 4 is described [105,106,107], while skewing in both progressive and non-progressive CLL patients towards a Th1 phenotype also has been reported, in which CD4^+^ T-cells produce more interferon y (IFNy), possibly contributing to an anti-tumor response [103,108].

Th2 and Th17 cells are increased in LN of FL patients [109,110]. Although absolute numbers of Th1 cells were small, high Th1 numbers correlated with rapid transformation [111]. Furthermore, CD4^+^ tumor-infiltrating T helper cells from FL patients showed a skewing towards an effector memory phenotype, while naive and central memory cells were decreased [110]. Decreases in circulating CD4^+^ T-cells were found in PB of FL patients, attributable to a decrease in naive T-cells [105]. Although CD8^+^ numbers were normal, a skewing towards T effector memory cells re-expressing CD45RA (Temra) cells could be observed within the CD8^+^ compartment [105].

T-cell skewing is observed in MZL as well. T-cell infiltrates in mucosa-associated lymphoid tissue (MALT) lymphoma predominantly have a Th1 phenotype, while in some cases CD8^+^ T-cells were observed as the main infiltrating T-cell type [112,113,114,115]. Interestingly, lower absolute numbers of circulating CD4^+^ T-cells were found in extranodal MZL with predominance of the naïve phenotype [105]. Similar to CLL, circulating CD8^+^ T-cells in MZL had a terminally differentiated phenotype [105].

It thus seems that T-cells from B-NHL patients are skewed towards a more antigen-experienced phenotype, and have a disturbed Th1/Th2 balance, although the latter varies between different B-NHL subtypes.

Skewing of T-cells can greatly influence T-cell therapy. Since memory T-cells are the driving force of T-cell persistence in vivo, and their presence is correlated with highly durable clinical remissions in CAR T-cell therapy, it is plausible that the composition of the patients’ T-cell subsets may influence the therapy outcome. For CAR T-cells, indeed, persistence and peak expansion of CAR T-cells in vivo are predictors of favorable clinical outcome in B-NHL, coinciding with the presence of memory-like T-cells [67,76,78,116]. This is further emphasized by the effect of the generation of CAR T-cells from different T-cell subsets, with CAR T-cells generated from central memory T-cells showing most favorable responses [117]. Additionally, predicting the clinical outcome of CAR T-cell therapy in CLL patients is possible by analyzing the apheresis product before generating CAR T-cells. Elevated numbers of memory-like T-cells, characterized by CD8^+^/CD27^+^/CD45RO^−^_,_ were found in the product in CLL patients that do respond to therapy [76]. Furthermore, CAR T-cell products from CLL patients that had elevated levels of memory-like CD8^+^ CAR T-cells negative for PD-1 correlated with improved clinical outcome [76]. For BsAb therapy, it has been implicated that effector memory T-cells are responsible for the activity [81]; however, this such a correlation could not be confirmed in BsAb trials in B-NHL.

### 3.3. Inhibitory Receptor Expression and Exhaustion in B-NHL

Chronic stimulation and inflammatory signals can induce exhaustion of T-cells leading to loss of effector function. Multiple exhaustion markers have been extensively studied, including PD-1, T-cell immunoglobulin, and mucin domain-containing protein 3 (TIM3), T-cell immunoglobulin and ITIM domain (TIGIT), CTLA-4, and lymphocyte activation gene-3 (LAG3), and negatively regulate T-cells via different mechanisms as extensively reviewed in [118]. Ligands for exhaustion markers on T-cells are often expressed on tumor cells or bystander cells in the TME and have been studied for DLBCL, CLL, FL, MCL and MZL.

In DLBCL, an estimated 11–24% of all patients show high expression of PD-L1 on tumor cells and high PD-1 expression on T-cells, which are associated with poor patient survival [116,119,120,121,122,123,124]. Additionally, PD-L1 expression on DLBCL cells correlates with PD-1 expression on T-cells [121]. Blocking PD-L1 promoted the proliferation and production of IFNy in T-cells demonstrated by an allogenic co-culture set-up [116]. Sufficient T-cell infiltration in DLBCL tumors may be required for successful anti-PD-L1/PD-1 therapy [125]. However, expression of PD-L1 and PD-1 may not always be responsible for the inhibitory effect in T-cells in DLBCL [125]. In addition, in some DLBCL cases, higher frequencies of CD4^+^TIM3^+^ and CD8^+^TIM3^+^ T-cells were observed [124,126], correlating with disease severity [124]. Additionally, T-cells in DLBCL may display increased expression of CD244 [127]. Furthermore, reduced expression of IFNy, tumor necrosis factor α (TNFα), and IL-2 was observed in intratumoral Tem cells in DLBCL, which co-expressed inhibitory receptor TIGIT and PD-1 [127]. Immunomodulatory receptor LAG3 and CTLA-4 may play lesser roles in DLBCL, as no differences in LAG3 expression on immune cells in the tumor microenvironment were observed compared to heathy donors [128]. Interestingly, CTLA-4 expression on T-cells in DLBCL may actually have a favorable prognosis [129].

Additionally, in CLL, exhaustion may play a role. T-cells derived from these patients display increased expression of PD-1, BTLA, CTLA-4, TIGIT, CD160 and CD244 [99,103,130,131,132,133]. However, production of cytokines by T-cells is similar to that of healthy controls, implying that T-cells in CLL do not reflect the classic exhausted phenotype despite expression of exhaustion marker [132]. In FL, TILs are shown to have increased expression of PD-1, TIM-3, TIGIT and LAG-3 [127,131,134,135,136,137,138,139]. In contrast to CLL, these T-cells often show reduced production of IFNy, IL-2, TNFα, granzyme B and perforin upon T-cell stimulation, indicating that these cells indeed might be exhausted [127,136,137,138,140]. Unlike other B-NHL subtypes, expression of PD-L1 on tumor cells was mostly absent in CLL and FL [141].

In MCL, intratumoral T-cells display significantly higher expression of TIGIT, CD244, and LAG3 compared to controls, especially in the effector-memory compartment [127]. Only a subset of MCL patients expressed the TIGIT ligand CD155 while no PD-L1 on tumor cells was detected. However, PD-L1 was expressed by intratumoral macrophages [127]. In contrast, others have shown that PD-L1 could be detected on MCL cells at both messenger RNA and protein level as well, although its receptor PD-1 was not significantly highly expressed on T-cells compared to healthy donors (HD) [142,143]. Conversely, another study has reported PD-L1 to be rarely expressed on MCL cells [144]. Thus, the role of PD-1/PD-L1-axis in MCL remains a subject of debate since many studies have reported opposing results.

In MZL, PD-1 and PD-L1 also might play a role. PD-L1 was expressed by splenic MZL cells [115], and its ligand PD-1 was found expressed on T-cells in extranodal MZL and MALT lymphoma [145,146].

Despite high expression of exhaustion markers on B-NHL-derived T-cells, the efficacy of ICB is disappointing, which may not only be explained by a relative low tumor mutational load (see Section 3.1) but also by low expression of inhibitory ligands such as PD-L1 on the tumor cells. In contrast, tumor cells in HL and PMBCL acquire a copy gain or amplification of 9p24.1 leading to aberrant expression of PD-L1 [147,148,149]. In PMBCL, high PD-L1 expression also correlated with PFS after pembrolizumab treatment. Thus, low expression of PD-L1 in B-NHL may in part explain the failure of ICB in these other B-NHL subtypes. However, ICB does not seem to resolve or prevent T-cell exhaustion on its own. ICB may only be successful in a subset of patients where expression of inhibitory ligands is high, as in PMBCL patients and a subset of DLBCL patients.

T-cell exhaustion can influence the efficacy of CAR T-cell therapy as well. For example, five patients with the highest PD-1/PD-L1 interactions showed no response to therapy, or quickly relapsed in a CAR T-cell trial for DLBCL patients [64]. In addition, 11 patients with the highest LAG3 levels also did not respond or relapsed [64]. Similar results were observed in another CAR T-cell trial in DLBCL patients [79]. CLL patients responding to CAR T-cell therapy had increased populations of PD-1 negative memory T-cells [76]. These results indicate, perhaps unsurprisingly, that T-cell exhaustion is a major cause of failure to CAR T-cell therapy.

It is currently unclear what role exhaustion plays in the treatment of patients with BsAbs since this has not been investigated extensively to our knowledge.

### 3.4. Functional Defects

Chronic stimulation of T-cells by tumors can lead to functional impairments such as defective cytokine production, activation, synapse formation and cytotoxic potential. For example, impaired formation of the immunological synapse impairs CAR T-cell efficacy, and therapy effectiveness can even be predicted by assessing the quality of synapse formation between the CAR T-cell and target cells [150]. The functionality of T-cells has mainly been investigated in CLL, FL and MZL.

In CLL, T-cells retain cytokine production, but have impaired synapse formation proliferation, activation, and cytotoxicity [136,151,152].

In FL, similar defects have been reported. Upon T-cell receptor (TCR) triggering, TILs in FL have a defective proliferative capacity [134,140], impaired motility as well as synapse formation [153,154]; the latter was also reported in transformed FL and de novo DLBCL [153]. Despite these dysfunctions, 3D confocal imaging revealed increased numbers of granzyme B positive CD8^+^ T-cells in FL samples compared to reactive lymph nodes [155]. These T-cells were shown to form synapses at the follicle border, implying that they can still be cytotoxic [155].

In MZL, induction of MALT lymphoma by *Helicobacter pylori* may impair T-cell functionality demonstrated by reduced killing of autologous Epstein–Barr virus B-cells [156].

It can thus be concluded that different B-NHL subtypes harbor different functional defects, although most seem to have acquired defective cytotoxic and proliferative capacities as well as impaired synapse formation. Nevertheless, it remains to be elucidated whether this is also the case for other B-NHL subtypes.

### 3.5. T-Cell Metabolism in B-NHL

It has been widely accepted that T-cell metabolism dictates function and development as extensively reviewed previously [151,152]. In short, naive T-cells utilize many different metabolites for their energy needs, while effector T-cells rely on glycolysis as the primary metabolic pathway, and memory T-cells rely on the oxidative phosphorylation of lipids [152]. It is important to note that access to these metabolites may be restricted as a result of competition by proliferating tumor cells in the tumor microenvironment [157]. Especially in the more aggressive forms of B-NHL, nutrient restriction may induce T-cell dysfunction. While lymphoma cells have high activity of oxidative phosphorylation or glycolysis (or both), care must be taken not to overestimate metabolic activity of B-NHL cells based on data derived from cell lines that may have adapted themselves to the nutrient-rich medium of in vitro cultures [158,159]. Currently, data on T-cell metabolism in DLBCL, FL, MCL, or MZL are lacking and most results regarding T-cell metabolism in B-NHL are derived from analysis of CLL patient material. In these data, it has been shown that T-cells have impaired expression of the glucose transporter Glut1, which coincides with reduced glucose uptake and mitochondrial defects, which impact CAR T cell therapy [160,161]. Thus, since lymphoma cells can exert metabolic pressure and metabolism is important for the effector function of T-cells, it is highly likely that reduced glycolysis and impaired mitochondrial fitness in T-cells contribute to the decreased efficacy of T-cell-based therapies in CLL, and possibly in DLBCL, FL, MCL, or MZL as well [151].

### 3.6. Regulatory T-Cells and T Follicular Helper Cells

Regulatory T-cells (Tregs), a subset of CD4^+^ T-cells that express forkhead box protein P3 (FOXP3), play a major role in preventing autoimmunity by dampening immune responses. However, Tregs have conversely been implicated in hindering effective anti-tumor immunity as well [162]. Tregs have been implicated to play a role in DLBCL, CLL, FL and MZL, possibly being responsible for suboptimal responses observed in T-cell mediated therapy.

Although Tregs are classified as immunosuppressive, increased numbers of Tregs correlate with favorable prognosis in DLBCL and FL, where low Treg numbers can predict transformation of FL to DLBCL [163,164,165,166,167,168,169,170]. For FL, it has been shown that these Tregs are suppressive as they inhibited proliferation and cytokine production of T-cells [166]. Tregs directly suppress proliferation of B-cells [171], and perhaps leukemic cells as well. Therefore, it is possible that Tregs inhibit the malignant FL or DLBCL clone, resulting in better prognosis. In CLL, many studies have shown that Treg compartments are expanded compared to controls, and correlate with advanced disease and number of circulating CLL cells [98,103,105,172,173,174]. Furthermore, it has been shown that in CLL, increased Treg numbers correlate with shorter time to first treatment, indicating a negative effect of these cells on tumor control [175]. In MZL, increased amounts of Tregs could be found in MALT lymphomas [176]. However, this could not be recapitulated for other forms of MZL [177]. The above mentioned observations indicate that Tregs play an important role in B-NHL, but their exact place in disease progression or regression is not well defined.

T follicular helper cells (Tfh), characterized as CD4^+^CXCR5^+^PD-1^+^_,_ can play a role in support of the TME and are well studied in DLBCL and FL. For DLBCL, Tfh cells seem to promote DLBCL cell growth and survival, via IL-10 secretion [178]. These DLBCL Tfh cells were found to secrete IL-10 in vast amounts [178], and therefore might also influence T-cell activation and proliferation, since these can be inhibited by IL-10 [179]. Another subset of Tfh cells are follicular Tregs (Tfr). Compared to conventional Tregs, follicular Tregs (Tfr) have similar functions, are identified by CD4^+^CXCR5^+^Foxp3^+^, and may regulate follicular T helper cells (Tfh) and B-cells (reviewed in [180]). In DLBCL, Tfr cells are enriched [178]. Tfr cells were able to inhibit autologous T-cell proliferation and IFNy production and support tumor cell growth [165]. Additionally, in CLL, Tfh cells are involved. It has been shown that CLL proliferation is induced by CD40 ligand (CD40L) and IL-21 stimulation, of which the latter is produced by Tfh cells [181]. Furthermore, in FL, Tfh cells play an important role in supporting survival of the cancer cells. Upregulation of the IL-4, TNF, and CD40L pathways in Tfh cells, expression of CD40L on Tfh cells, as well as secretion of IL-4 and IL-21, and low secretion of IL-17 have been shown to support survival of FL cells [109,182,183,184]. IL-4 and CD40L signaling can lead to production of CCL17 and CCL22 by the malignant B-cells [183]. This, in turn, can again lead to migration of Tregs and IL-4 secreting T-cells, resulting in a self-sustaining positive feedback loop [183]. It thus seems that Tfh cells can promote lymphomas via direct support of the tumor cells as well as negatively influencing TIL and therefore may possibly influence the outcome of T-cell-based therapy.

## 4. Possible Solutions

The multiple levels of T-cell dysfunction that we describe here could serve as novel targets to improve the efficacy of T-cell-based therapies. Of the many approaches that have been described, we will focus on three possible solutions that could already be tested within clinical trials. The first is the addition of agents that target T-cells to improve their efficacy, the second is timing of autologous T-cell-based therapy and the third are specific solutions to improve CAR T efficacy either by adjusting the CAR T-cell design itself, or the CAR T cell production process.

### 4.1. Combination Therapy Improves ICB and BsAbs and Potentially CARs

Lenalidomide is an immunomodulatory drug that has been approved for treatment of multiple myeloma, MCL, and myelodysplastic syndromes. Lenalidomide was shown to bind to the E3 ubiquitin ligase cereblon, thereby inducing degradation of the transcription factors Aiolos and Ikaros [185,186]. Since Aiolos and Ikaros regulate T-cell fate, lenalidomide treatment induces skewing to a Th1 phenotype with enhanced expression of IL-2 and IFNy as well as inhibition of proliferation and functioning of Tregs [186,187,188]. In line with this, treatment with lenalidomide also had a positive effect on T-cells from patients with different B-cell malignancies. In CLL, FL and DLBCL, it has been shown that lenalidomide reverses dysfunctional synapse formation and improves CLL T-cell motility [151,157,189]. Furthermore, it has been shown that treatment of FL peripheral blood mononuclear cells (PBMCs) with lenalidomide resulted in enhanced activation, proliferation, and production of IFNy and IL-2 by T-cells upon TCR stimulation [190]. Additionally, avadomide, a cereblon E3 ligase modulator similar to lenalidomide, improved T-cell responses in CLL via type I and II interferon signaling, eventually leading to responses in patient xenograft models with ICB [191].

Since T-cells from patients with B-cell malignancies might benefit from lenalidomide treatment, combination strategies involving T-cell therapy might therefore be feasible. These types of agents have already been tested in a clinical setting. For DLBCL, a phase II trial was started to evaluate the efficacy of CD19 CAR in combination with either lenalidomide or rituximab (NCT04002401). The studies on the combination of lenalidomide with either ICB or BsAbs in B-NHL are still limited but do seem to show enhanced effects [38,39,40,51]. Furthermore, reports have shown that for multiple myeloma, the addition of lenalidomide to BsAbs or ICB can be beneficial [192,193].

Results with lenalidomide combinations show that targeted drugs can potentiate T-cells and thereby improve T-cell-based therapy. However, due to toxicities, other options in addition to lenalidomide need to be considered. Idelalisib might be a feasible candidate. Especially for CLL, reports have been published regarding this phosphoinositide 3-kinase (PI3K) δ inhibitor. Recently, it has been shown that idelalisib inhibits IFNy production and skews HD T-cells towards more effector differentiation upon stimulation [194]. In CLL, idelalisib is associated with a decreased amount of Tregs [189]. Furthermore, it led to a decrease in effector CD8^+^ T-cells together with decreased IFNy production and degranulation in the Eµ-TCL1 mouse model [195]. Whether decreased Tregs and IFNy production of T-cells in CLL are beneficial for T-cell mediated therapy still needs to be elucidated. Nevertheless, a phase II trial combining pembrolizumab and idelalisib in CLL is currently being conducted (ClinicalTrials.gov Identifier: NCT02332980). In DLBCL, PBMC stimulation in the presence of idelalisib also resulted in less production of IFNy, but more IL-2 and a less differentiated phenotype as measured by CD27 and CD28 expression [196], which might be beneficial. Adoptive transfer of DLBCL-derived T-cells treated with idelalisib also showed increased in vivo persistence [196].

Additionally, ibrutinib has gained much interest with regard to its use in combination with autologous T-cell therapy. In addition to targeting BTK, ibrutinib also inhibits IL-2-inducible T-cell kinase (ITK) expressed by T-cells [197]. ITK inhibition by ibrutinib was shown to induce Th1 skewing in healthy donor T-cells [197]. However, it has previously been shown that ITK knockdown leads to increased Treg and Th1 differentiation [198,199], and ITK deficiency or inhibition decreased degranulation of CD8^+^ T-cells resulting in impaired cytotoxicity [200]. It thus seems that specific ITK inhibition may not lead to improved T-cell responses as seen upon ibrutinib treatment, and the observed improved T-cell function may be a subsequent result of decreased CLL function and presence induced by ibrutinib. Effects of ibrutinib on T-cells in B-cell malignancies have been well described for CLL. Ibrutinib induces Th1 skewing, together with CTLA-4 and PD-1 downregulation, a decreased Treg/CD4 ratio, and an increased T-cell receptor repertoire [197,201,202,203]. T-cells of CLL patients that had received prior ibrutinib had improved cytotoxic activity upon treatment with either a CD3xCD19 or CD3xROR1 BsAb, possibly due to enhanced synapse formation [59,62]. Efficacy of ICB might also be enhanced in combination with ibrutinib. In a Eµ-TCL1 CLL mouse model, it has been demonstrated that anti-PD-1 or PD-L1 in combination with ibrutinib led to increased tumor control [204]. In addition, in a mice xenograft MCL model, ibrutinib enhanced survival and ameliorated expression of exhaustion and inhibitory receptors on CAR T-cells [205]. Additionally, in a mouse lymphoma model, which was resistant to ibrutinib monotherapy, the combination of ibrutinib with anti-PD-L1 therapy significantly improved survival compared to anti-PD-L1 therapy alone [206]. Despite these promising results in a mouse model, results from a phase I/IIa study combining ibrutinib with nivolumab for CLL, FL and DLBCL were rather disappointing since the efficacy of the combination was comparable to monotherapy with either ibrutinib or nivolumab [41]. Similar results were obtained for FL and DLBCL upon combining ibrutinib with anti-PD-L1 antibody durvalumab [45].

Long-term treatment with ibrutinib, idelalisib or lenalidomide can lead to the development of severe adverse events [207,208]. Therefore, adequate timing and administering the correct dose of the specified therapy is important. For example, prior to starting T-cell-based therapy, patients might undergo induction with an immunomodulatory agent to restore T-cell dysfunction, which may boost subsequent T-cell-based therapies in return. Unfortunately, to our knowledge, no (pre)clinical data are currently available that support the beneficial effects of differential timing of therapy to improve T-cell-based therapy.

In conclusion, T-cell modulatory agents are readily available and may be able to enhance T-cell therapy in a combination setting, in which timing could also play a role in the future. Lenalidomide trials, especially, indicate that they improve T-cell-based therapy, but future studies are needed to confirm this.

### 4.2. Consolidation Therapy

As discussed above, suppression of T-cell function can be the result of overwhelming numbers of tumor cells causing metabolic pressure in addition to restriction of T-cell function by expression of inhibitory ligands on tumor cells. Therefore, it may be beneficial to debulk tumor cells prior to T-cell-based therapies as it may create a window of opportunity in which T-cells recover (all or part of) their functions. This was perhaps unintentionally demonstrated in the first CAR T-cell trial where two patients who achieved CR after cytoreductive chemotherapy sustained that CR after subsequently receiving CAR T-cells [66]. Additionally, treatment of CLL patients with venetoclax and obinutuzumab not only led to eradication of the CLL, but also to reduced Tregs, Tfh and PD-1 expression on CD8^+^ T-cells, indicating a reversion of T-cell dysfunction and recovery of T-cell skewing [104]. Blinatumomab as consolidation after rituximab and chemotherapy in 28 DLBCL patients led to an ORR of 89% [52], which is slightly improved in comparison to R-CHOP alone [209], although it has to be awaited whether PFS increases upon addition of blinatumomab. These results indicate that T-cell therapy as a consolidation therapy might be a good alternative strategy to improve the outcome of patients. However, one of the difficulties of consolidation therapy is the timing of the treatments. T-cell-based therapies rely on the availability of the antigen and therefore may be applied as consolidation prior to reaching minimal residual disease negativity to achieve full efficacy.

### 4.3. Improving CAR T-Cell Therapy

Persistence and peak expansion of CAR T-cells are predictors of favorable clinical outcome and can greatly influence CAR T-cell efficacy [67,76,78,210]. Numerous methods to improve persistence and expansion are available and are currently investigated and focus primarily on increasing the numbers of memory T-cells. The phenotype of expanded CAR T-cell products in CLL patients had a more differentiated phenotype, becoming the most apparent after 20 days of culturing [211], and may impact the generated CAR T cell product [76]. However, failure to generate adequate CAR T-cells could not be rescued by IL-2 or IL-7/IL-15 supplementation in CLL patients to levels to that of HD, implying that different methods to generate memory CAR T cells are necessary [211,212]. The phenotype of CARs generated from ALL patients were highly similar to CARs generated from HDs, which may explain why the clinical effectiveness of CAR T-cell therapy in ALL is high [213]. These results indicate that enhancing the memory phenotype of CAR T-cells may benefit efficacy of the therapy.

The type of costimulatory ligand in CAR can greatly impact CAR T cell function and phenotype. For example, differences in phenotype between 28z and BBz have been demonstrated in animal models, which show that the 4-1BB signaling domain enhanced CAR T-cell memory development and persistence, and promoted oxidative phosphorylation and mitochondrial biogenesis, while the CD28 signaling domain pushed towards an effector phenotype and glycolysis [214,215]. Selecting the correct co-stimulatory domain, or combining multiple co-stimulatory domains, may therefore improve CAR T cell function. Indeed, a combination of 4-1BB and inducible T cell co-stimulator (ICOS) into one CAR design greatly enhanced persistence and tumor eradication compared to either 4-1BB or ICOS alone in a mouse xenograft model bearing pancreatic tumors [216]. However, it may not be necessary to alter the CAR T cell design per se. Other methods exist to improve CAR T cell persistence and memory formation without altering the CAR T cell design itself.

The most evident method to obtain persisting CAR T cells may be through generating CAR T cells from a pool of purified memory T-cells. Wang et al. treated eight patients with first generation central memory-derived CAR T-cell infusions, and eight more patients received second generation central memory-derived 28z CAR T-cells [86]. The CR rate was 38% (three patients) for patients receiving first generation CAR T-cells, and the CR rate was 75% (six patients) for patients receiving a second generation CAR T-cell product [86]. Cytokines, metabolites, or inhibitors can be added during the generation of CAR T-cells to skew the T-cell composition towards a memory phenotype. For example, IL-15 added during CAR T-cell generation can preserve memory phenotype [210], although IL-15 supplementation on its own may not be enough to completely enhance CAR T cell efficacy [211,212]. In addition, metabolites such as L-arginine can also be added during culture. This has been shown to promote T-cell survival and oxidative phosphorylation and increase the percentage of central memory T-cells in a mouse model [217]. As a result, tumor-bearing mice injected with L-arginine-treated CAR T-cells had increased survival over the control group [217]. Additionally, the PI3K-inhibitor idelalisib can be added to CAR T-cells culture to enhance representation of naïve-like CD45RA^+^CCR7^+^ subset, and simultaneously reduce expression of inhibitory receptors PD-1 and TIM-3 [212]. Idelalisib could enhance the representation of memory-like T-cells in HD and CLL; however, the representation of memory-like T-cells in CLL in idelalisib-treated samples was still much lower compared to HD. Nevertheless, CAR T-cells generated with idelalisib slightly outperformed non-idelalisib cultured CAR T-cells in mice [212]. Improving memory formation can also be established by preventing lysosomal degradation of the CAR, after recognizing its cognate antigen [218]. Prevention of ubiquitination of CARs targeted for degradation in lysosomes not only improved the expression of surface CAR, but also increased the lysing capabilities of the CAR T-cells, absolute counts, and percentage of central memory T-cells [218]. Furthermore, the metabolic phenotype of these cells corresponded with that of memory T-cells, such as having improved oxygen consumption rate, maximum respiration, and spare respiratory capacity, which indicate enhanced oxidative phosphorylation and mitochondrial biogenesis [218]. These methods not only describe the many ways in which T-cells can be pushed towards a memory phenotype, but also demonstrate the increased CAR T efficacy as a result.

Preventing T-cell exhaustion either as a result of chronic stimulation, or indirectly induced by cancer cells, may improve CAR T-cell therapy as well. Overexpression of c-Jun, a component of the AP-1 transcription factor, can protect CAR T-cells from exhaustion and greatly enhance proliferation compared to a control CAR [219]. Additionally, overexpression of c-Jun greatly enhanced efficacy in solid tumors and improved survival in mice bearing osteosarcomas [219]. Alternatively, CAR T-cells can be equipped with a PD-1 dominant negative receptor to prevent signaling of exhaustion. Such CARs outperformed control CARs in a murine mesothelin mouse model [220]. Prevention of exhaustion may especially be beneficial in tumors where the PD-L1/PD-1 axis is important in tumor cell survival.

## 5. Conclusions

This review comprehensively shows that the efficacy of autologous-based T-cell therapy differs among B-NHL subtypes as well as per treatment modality. ICB has shown very discouraging results, and even in combination strategies, efficacy does not seem to improve and ICB is therefore likely not a feasible treatment modality in B-NHL including CLL. This is in contrast to BsAbs and CAR T-cells, which do elicit responses, although not to the extent of those observed in ALL. These diminished responses are likely due to different aspects of T-cell dysfunction that have been described in B-NHL subtypes. The recent advantages made in CAR T-cell production, and the development of immunomodulatory agents can be used to overcome T-cell dysfunction in these malignancies and improve T-cell mediated therapy for B-NHL.

## Figures and Tables

**Table 1:** Reported studies of immune checkpoint blockade in B-cell non-Hodgkin lymphoma (B-NHL).

Study	Treatment	Phase	Disease	N	CR	ORR	Trial ID (NCT)
O’Mahony et al., 2007 [20]	Ipilimumab	II	FL	2	0/2 (0%)	1/2 (50%)	N/A
			MCL	2	0/2 (0%)	1/2 (50%)	
Berger et al., 2008 [21]	Pidilzumab	I	CLL	3	0/3 (0%)	0/3 (0%)	N/A
			DLBCL	2	0/3 (0%)	0/2 (0%)	
			FL	1	1/1 (100%)	1/1 (100%)	
Ansell et al., 2009 [23]	Ipilimumab	I	FL	14	0/14 (0%)	1/14 (7%)	NCT00089076
			DLBCL	3	1/3 (33%)	1/3 (33%)	
			MCL	1	0/1 (0%)	0/1 (0%)	
Bashey et al., 2009 [22]	Ipilimumab	I	MCL	1	0	1/1 (100%)	NCT00060372
			CLL	2	0	0/2 (0%)	
Westin et al., 2014 [31]	Pidilizumab and rituximab	II	FL	29	15/29 (52%)	19/29 (66%)	NCT00904722
Ansell et al., 2016 [29]	Nivolumab and ipilimumab	Ib	B-NHL	15	0/15 (0%)	3/15 (20%)	NCT01592370
Lesokhin et al., 2016 [26]	Nivolumab	Ib	FL	10	1/10 (10%)	4/10 (40%)	NCT01592370
			MCL	4	0/4 (0%)	0/4 (0%)	
			MZL	1	0/1 (0%)	0/1 (0%)	
			DLBCL	11	2/11 (18%)	4/11 (36%)	
Ding et al., 2017 [25]	Pembrolizumab	II	CLL	16	0/16 (0%)	0/16 (0%)	NCT02332980
			CLL with RT	9	1/9 (11%)	4/9 (44%)	
Ding et al., 2017 [24]	Pembrolizumab	II	FL	18	0/18 (0%)	2/18 (11%)	NCT02332980
			MZL	2	0/2 (0%)	0/2 (0%)	
Nastoupil et al., 2017 [30]	Pembrolizumab and rituximab	II	FL	25	12/25 (48%)	16/25 (64%)	NCT02446457
Younes et al., 2017 [36]	Atezolizumab, Obinutuzumab and Bendamustin	Ib/II	FL	15	10/15 (67%)	12/15 (80%)	NCT02596971
Ansell et al., 2019 [27]	Nivolumab	II	auto-HCT failed DLBCL	87	3/87 (3%)	9/87 (10%)	NCT02038933
			auto-HCT ineligible DLBCL	34	0/34 (0%)	1/34 (3%)	
Khouri et al., 2018 [40]	Ipilimumab and lenalidomide	II	FL (allo-HSCT)	2	1/2 (50%)	2/2 (100%)	NCT01919619
			MCL (allo-HSCT)	3	2/3 (67%)	3/3 (100%)	
			DLBCL (allo-HSCT)	1	0/1 (0%)	1/1 (100%)	
			CLL (allo-HSCT)	2	0/2 (0%)	0/2 (0%)	
			MCL (auto-HSCT)	2	2/2 (100%)	2/2 (100%)	
			FL (auto-HSCT)	1	1/1 (100%)	1/1 (100%)	
Palomba et al., 2017 [35]	Atezolizumab and obinutuzumab	Ib	FL	26	N/A	57%	NCT02220842
			DLBCL	23	N/A	16%	
Younes et al., 2018 [37]	Atezolizumab, Rituximab and CHOP	I/II	DLBCL	40	31/40 (78%)	35/40 (88%)	NCT02596971
Armand et al., 2019 [28]	Pembrolizumab Pembrolizumab	Ib II	PMBCL PMBCL	21 53	7/21 (33%) 7/53 (13%)	48% 45%	NCT01953692 NCT02576990
Bond et al., 2019 [38]	Nivolumab and lenalidomide	I	B-cell lymphoma *	10	1/10 (10%)	3/10 (30%)	NCT03015896
Casulo et al., 2019 [39]	Durvalumab monotherapy or in combination with lenalidomide ± rituximab or rituximab ± bendamustine	I/II	DLBCL	38	3/38 (8%)	7/38 (18%)	NCT02733042
			FL	22	6/22 (27%)	13/22 (59%)	
Younes et al., 2019 [41]	Nivolumab and ibrutinib	I/IIa	CLL/SLL	36 (30 CLL)	0/36 (0%)	22/36 (61%)	NCT02329847
			RT	20	2/20 (10%)	13/20 (65%)	
			FL	40	4/40 (10%)	13/40 (33%)	
			DLBCL	45	7/45 (16%)	16/45 (36%)	
Zinzani et al., 2019	Nivolumab and brentuximab vedotin	I/II	PMBCL	30	11/30 (37%)	22/30 (73%)	NCT02581631
Frigault et al., 2020 [47]	Pembrolizumab after auto-SCT	II	DLBCL	29	17/29 (59%)	17/29 (59%)	NCT02362997
Herrera et al., 2020 [45]	Durvalumab and ibrutinib	Ib/II	FL GCB DLBCL Non-GCB DLBCL	27 16 16	1/27 (4%) 1/16 (6%) 5/16 (31%)	7/27 (26%) 2/16 (13%) 6/16 (38%)	NCT02401048

*N* = number of patients, CR = complete response, ORR = overall response rate, N/A = not available, CLL = chronic lymphocytic leukemia, DLBCL = diffuse large B-cell lymphoma, MZL = marginal zone lymphoma, FL = follicular lymphoma, MCL = mantle cell lymphoma, SLL = small lymphocytic leukemia, PMBCL = primary mediastinal large B-cell lymphoma, RT = Richter’s transformation, CHOP = cyclophosphamide, hydroxydaunorubicin, vincristine and prednisone, auto-HSCT = autologous hematopoietic stem cell transplantation, allo-HSCT = allogeneic hematopoietic stem cell transplantation, GCB = Germinal center B-cell, * 5 patients were diagnosed with DLBCL.

**Table 2:** Reported studies of bispecific antibodies in B-NHL.

Study	Treatment	Phase	Disease	N	CR	ORR	Trial ID (NCT)
Goebeler et al., 2016 [48]	Blinatumomab	1	FL	15 *	6/15 (40%)	12/15 (80%)	NCT00274742
			MCL	7 *	3/7 (43%)	5/7 (71%)	
			DLBCL	11 *	4/11 (36%)	6/11 (55%)	
Viardot et al., 2016 [49]	Blinatumomab	2	DLBCL	21	4/21 (19%)	9/21 (43%)	NCT01741792
Bannerji et al., 2017 [55]	REGN1979 (CD3xCD20)	1	DLBCL	22	0/22 (0%)	4/22 (18%)	NCT02290951
			FL	11	0/11 (0%)	4/11 (36%)	
			MZL	2	0/2 (0%)	1/2 (50%)	
			MCL	2	0/3 (0%)	1/3 (33%)	
Katz et al., 2019 [52]	Blinatumomab after R-CHOP	2	DLBCL	28	NA	25/28 (89%)	NCT03023878
Morschhauser et al. 2019 [57]	Glofitamab and obinutuzumab	-	B-NHL ** (all included)	21	9/21 (43%)	10/21 (48%)	NCT03075696
			B-NHL ** (highest glofitamab dose)	10	8/10 (80%)	9/10 (90%)	
Poh et al., 2019 [51]	Blinatumomab and lenalidomide	1	B-NHL	18	50%	83%	NCT02568553
Schuster et al., 2019 [53]	Mosunetuzumab	1/1b	indolent B-NHL ***	64	27/64 (42%)	41/64 (64%)	NCT02500407
			aggressive B-NHL ***	119	22/119 (18%)	41/119 (34%)	
Bannerji et al., 2020 [56]	REGN1979	1	FL (≥5 mg)	28	21/28 (75%)	26/28 (93%)	NCT02290951
			FL (≥80 mg)	16	11/16 (69%)	15/16 (94%)	
			DLBCL (no prior CAR, ≥5 mg)	30	9/30 (30%)	14/30 (47%)	
			DLBCL (no prior CAR, ≥80 mg)	10	6/10 (60%)	6/10 (60%)	
			DLBCL (relapse after CAR, ≥5 mg)	23	5/23 (22%)	7/23 (30%)	
			DLBCL (relapse after CAR, ≥80 mg)	21	5/21 (24%)	7/21 (33%)	
Coyle et al., 2020 [50]	Blinatumomab	2	B-NHL ****	41	9/41 (22%)	15/41 (37%)	NCT02910063
Hutchings et al., 2020 [54]	Subcutaneous epcoritamab (GEN3013; CD3xCD20)	1/2	DLBCL/HGBCL	9	4/9 (44%)	5/9 (56%)	NCT03625037
			FL	6	0/6 (0%)	6/6 (100%)	

*N* = number of patients, CR = complete response, ORR = overall response rate, N/A = not available, CLL = chronic lymphocytic leukemia, DLBCL = diffuse large B-cell lymphoma, MZL = marginal zone lymphoma, FL = follicular lymphoma, MCL = mantle cell lymphoma, B-NHL = B-cell non-Hodgkin lymphoma, HGBCL = high-grade B-cell lymphoma, NA = not available. * *n* reflects only the number of patients that reached the target dose at 60 μg/m^2^/day, ** include DLBCL, primary mediastinal large B-cell lymphoma, MCL, RT, and (transformed) FL,*** indolent NHL mainly included FL. Aggressive B-NHL mainly includes DLBCL and transformed FL, **** 34 of 41 DLBCL, other 7 patients are classified as other lymphomas.

**Table 3:** Chimeric antigen receptor therapy clinical studies.

Study	CAR type	Phase	Disease	N	CR	ORR	Trial ID (NCT)
First Generation CAR							
Till et al., 2008 [66]	anti-CD20	I	FL	7	2/7 (29%)	3/7 (43%)	NCT00012207
Second Generation 28z CAR							
Brentjens et al., 2011 [69]	anti-CD19	I/II	CLL	7	0/7 (0%)	0/7 (0%)	NCT00466531
Kochenderfer et al., 2012 [67]	anti-CD19	I/II	FL	3	0/3 (0%)	3/3 (100%)	NCT00924326
			CLL	4	1/4 (25%)	3/4 (75%)	
Kochenderfer et al., 2013 [68]	anti-CD19	I	CLL	4	0/4 (0%)	0/4 (0%)	NCT01087294
			DLBCL	2	0/2 (0%)	0/2 (0%)	
			MCL	4	0/4 (0%)	2/4 (50%)	
Kochenderfer et al., 2015 [70]	anti-CD19	I/II	CLL	4	3/4 (75%)	4/4 (100%)	NCT00924326
			MZL	1	0/1 (0%)	1/1 (100%)	
			DLBCL	4	2/4 (50%)	4/4 (50%)	
Ramos et al., 2016 [72]	anti-k light chain	I	CLL	2	0/2 (0%)	0/2 (0%)	NCT00881920
			DLBCL	2	0/2 (0%)	0/2 (0%)	
			MCL	1	0/1 (0%)	0/1 (0%)	
Wang et al., 2016 [86]	anti-CD19 (1st gen)	I/II	DLBCL	7	2/7 (29%)	3/7 (43%)	NCT01318317
			MCL	1	1/1 (100%)	1/1 (100%)	
	anti-CD19 (2nd gen)	I	DLBCL	4	3/4 (75%)	3/4 (75%)	NCT01815749
			MCL	4	3/4 (75%)	3/4 (75%)	
Kochenderfer et al., 2017 [71]	anti-CD19	I/II	DLBCL	19	11/19 (58%)	15/19 (79%)	NCT00924326
			FL	2	2/2 (100%)	2/2 (100%)	
			MCL	1	1/1 (100%)	1/1 (100%)	
Geyer et al., 2018 [73]	anti-CD19	I	CLL *	8	2/8 (25%)	2/8 (25%)	NCT01416974
Locke et al., 2018 [74]	anti-CD19	I/II	DLBCL	101	59/101 (58%)	84/101 (83%)	NCT02348216
Jacobson et al., 2020 [75]	anti-CD19	II	FL	80	64/80 (80%)	76/80 (95%)	NCT03105336
			MZL	7	5/7 (71%)	6/7 (86%)	
Wang et al., 2020 [65]	anti-CD19	II	MCL	60	40/60 (67%)	56/60 (93%)	NCT02601313
Second Generation BBz CAR							
Kalos et al., 2011 [87]	anti-CD19	I	CLL	3	2/3 (67%)	3/3 (100%)	NCT00295477
		II					NCT00622232
Porter et al., 2015 [78]	anti-CD19	I	CLL	14	4/14 (29%)	8/14 (57%)	NCT01029366
Fraietta et al., 2016 [78]	anti-CD19	II	CLL	3	1/3 (33%)	3/3 (100%)	NCT01747486
		I/II					NCT01105247
		I/II					NCT01217749
Schuster et al., 2017 [79]	anti-CD19	I	FL	14	6/14 (42%)	7/14 (50%)	NCT02030834
			DLBCL	14	10/14 (71%)	11/14 (79%)	
Fraietta et al., 2018 [76]	anti-CD19	I	CLL	41	8/41 (20%)	16/41 (39%)	NCT01029366
		II					NCT01747486
		I					NCT02640209
Abramson et al., 2019 [81]	anti-CD19	I	DLBCL	255	135/255 (53%)	186/255 (73%)	NCT02631044
Cao et al., 2019 [85]	anti-CD19 **	N/A	DLBCL	10	5/10 (50%)	9/10 (90%)	ChiCTR-ONN-16009862, ChiCTR1800019288
Hirayama el al., 2019 [77]	anti-CD19	I/II	FL	8	7/8 (88%)	7/8 (88%)	NCT01865617
			FL ***	13	6/13 (46%)	6/13 (46%)	
Schuster et al., 2019 [64]	anti-CD19	II	DLBCL	93	40/93 (43%)	52/93 (56%)	NCT02445248
Siddiqi et al., 2019 [83]	anti-CD19	I/II	CLL	22	10/22 (64%)	18/22 (82%)	NCT03331198
Ying et al., 2019 [82]	anti-CD19 (4-1BB or CD28)	I/II	MZL	1	1/1 (100%)	1/1 (100%)	NCT03528421
			FL	5	5/5 (100%)	5/5 (100%)	
			DLBCL	2	1/2 (50%)	1/2 (50%)	
Gauthier et al., 2020 [88]	anti CD19	I/II	CLL ****	19	N/A	14/17 (88%)	NCT01865617
			CLL	19	N/A	10/19 (56%)	

CAR = chimeric antigen receptor, *N* = number of patients, CR = complete response, ORR = overall response rate, N/A = not available, CLL = chronic lymphocytic leukemia, DLBCL = diffuse large B-cell lymphoma, MZL = marginal zone lymphoma, FL = follicular lymphoma, MCL = mantle cell lymphoma, n/a = not available. * Patients were selected based on minimal residual disease (MRD) after purine-based chemotherapy. ** Patients received nivolumab during treatment. *** Transformed FL. **** Ibrutinib treated.

## References

[1] Armitage J.O., Gascoyne R.D., Lunning M.A., Cavalli F. (2017). Non-Hodgkin lymphoma. Lancet.

[2] Hallek M. (2019). Chronic lymphocytic leukemia: 2020 update on diagnosis, risk stratification and treatment. Am. J. Hematol..

[3] Morschhauser F., Flinn I.W., Advani R., Sehn L.H., Diefenbach C., Kolibaba K., Press O.W., Salles G., Tilly H., Chen A.I. (2019). Polatuzumab vedotin or pinatuzumab vedotin plus rituximab in patients with relapsed or refractory non-Hodgkin lymphoma: Final results from a phase 2 randomised study (ROMULUS). Lancet Haematol..

[4] Palanca-Wessels M.C.A., Czuczman M., Salles G., Assouline S., Sehn L.H., Flinn I., Patel M.R., Sangha R., Hagenbeek A., Advani R. (2015). Safety and activity of the anti-CD79B antibody–drug conjugate polatuzumab vedotin in relapsed or refractory B-cell non-Hodgkin lymphoma and chronic lymphocytic leukaemia: A phase 1 study. Lancet Oncol..

[5] Jacobsen E.D., Sharman J.P., Oki Y., Advani R.H., Winter J.N., Bello C.M., Spitzer G., Palanca-Wessels M.C., Kennedy D.A., Levine P. (2015). Brentuximab vedotin demonstrates objective responses in a phase 2 study of relapsed/refractory DLBCL with variable CD30 expression. Blood.

[6] Furman R.R., Cheng S., Lu P., Setty M., Perez A.R., Guo A., Racchumi J., Xu G., Wu H., Ma J. (2014). Ibrutinib Resistance in Chronic Lymphocytic Leukemia. N. Engl. J. Med..

[7] Bose P., Gandhi V.V., Konopleva M.Y. (2017). Pathways and mechanisms of venetoclax resistance. Leuk. Lymphoma.

[8] Hamid O., Robert C., Daud A., Hodi F.S., Hwu W.-J., Kefford R., Wolchok J.D., Hersey P., Joseph R.W., Weber J.S. (2013). Safety and Tumor Responses with Lambrolizumab (Anti–PD-1) in Melanoma. N. Engl. J. Med..

[9] Topalian S.L., Hodi F.S., Brahmer J.R., Gettinger S.N., Smith D.C., McDermott D.F., Powderly J.D., Carvajal R.D., Sosman J.A., Atkins M.B. (2012). Safety, Activity, and Immune Correlates of Anti–PD-1 Antibody in Cancer. N. Engl. J. Med..

[10] Meti N., Esfahani K., Johnson N.A. (2018). The Role of Immune Checkpoint Inhibitors in Classical Hodgkin Lymphoma. Cancers.

[11] Kantarjian H.M., Stein A., Gökbuget N., Fielding A.K., Schuh A.C., Ribera J.-M., Wei A., Dombret H., Foà R., Bassan R. (2017). Blinatumomab versus Chemotherapy for Advanced Acute Lymphoblastic Leukemia. N. Engl. J. Med..

[12] Topp M.S., Gökbuget N., Stein A.S., Zugmaier G., O’Brien S., Bargou R.C., Dombret H., Fielding A.K., Heffner L., Larson R.A. (2015). Safety and activity of blinatumomab for adult patients with relapsed or refractory B-precursor acute lymphoblastic leukaemia: A multicentre, single-arm, phase 2 study. Lancet Oncol..

[13] Kuwana Y., Asakura Y., Utsunomiya N., Nakanishi M., Arata Y., Itoh S., Nagase F., Kurosawa Y. (1987). Expression of chimeric receptor composed of immunoglobulin-derived V resions and T-cell receptor-derived C regions. Biochem. Biophys. Res. Commun..

[14] Eshhar Z., Waks T., Gross G., Schindler D.G. (1993). Specific activation and targeting of cytotoxic lymphocytes through chimeric single chains consisting of antibody-binding domains and the gamma or zeta subunits of the immunoglobulin and T-cell receptors. Proc. Natl. Acad. Sci. USA.

[15] Krause A., Guo H.-F., Latouche J.-B., Tan C., Cheung N.-K.V., Sadelain M. (1998). Antigen-dependent CD28 Signaling Selectively Enhances Survival and Proliferation in Genetically Modified Activated Human Primary T Lymphocytes. J. Exp. Med..

[16] Imai C., Mihara K., Andreansky M., Nicholson I.C., Pui C.-H., Geiger T.L., Campana D. (2004). Chimeric receptors with 4-1BB signaling capacity provoke potent cytotoxicity against acute lymphoblastic leukemia. Leuk..

[17] Maher J., Brentjens R.J., Gunset G., Rivière I., Sadelain M. (2002). Human T-lymphocyte cytotoxicity and proliferation directed by a single chimeric TCRζ /CD28 receptor. Nat. Biotechnol..

[18] Maude S.L., Teachey D.T., Porter D.L., Grupp S.A. (2015). CD19-targeted chimeric antigen receptor T-cell therapy for acute lymphoblastic leukemia. Blood.

[19] Brentjens R.J., Davila M.L., Riviere I., Park J., Wang X., Cowell L.G., Bartido S., Stefanski J., Taylor C., Olszewska M. (2013). CD19-Targeted T Cells Rapidly Induce Molecular Remissions in Adults with Chemotherapy-Refractory Acute Lymphoblastic Leukemia. Sci. Transl. Med..

[20] O’Mahony D., Morris J.C., Quinn C., Gao W., Wilson W.H., Gause B., Pittaluga S., Neelapu S., Brown M., Fleisher T.A. (2007). A Pilot Study of CTLA-4 Blockade after Cancer Vaccine Failure in Patients with Advanced Malignancy. Clin. Cancer Res..

[21] Berger R., Rotem-Yehudar R., Slama G., Landes S., Kneller A., Leiba M., Koren-Michowitz M., Shimoni A., Nagler A. (2008). Phase I Safety and Pharmacokinetic Study of CT-011, a Humanized Antibody Interacting with PD-1, in Patients with Advanced Hematologic Malignancies. Clin. Cancer Res..

[22] Bashey A., Medina B., Corringham S., Pasek M., Carrier E., Vrooman L., Lowy I., Solomon S.R., Morris L.E., Holland H.K. (2009). CTLA4 blockade with ipilimumab to treat relapse of malignancy after allogeneic hematopoietic cell transplantation. Blood.

[23] Ansell S.M., Hurvitz S.A., Koenig P.A., LaPlant B.R., Kabat B.F., Fernando D., Habermann T.M., Inwards D.J., Verma M., Yamada R. (2009). Phase I Study of Ipilimumab, an Anti-CTLA-4 Monoclonal Antibody, in Patients with Relapsed and Refractory B-Cell Non-Hodgkin Lymphoma. Clin. Cancer Res..

[24] Ding W., Laplant B., Witzig T.E., Johnston P.B., Colgan J.P., Rech K.L., Leis J.F., Feldman A.L., He R., Nowakowski G.S. (2017). PD-1 Blockade with Pembrolizumab in Relapsed Low Grade Non-Hodgkin Lymphoma. Blood.

[25] Ding W., LaPlant B.R., Call T.G., Parikh S.A., Leis J.F., He R., Shanafelt T.D., Sinha S., Le-Rademacher J., Feldman A.L. (2017). Pembrolizumab in patients with CLL and Richter transformation or with relapsed CLL. Blood.

[26] Lesokhin A.M., Ansell S.M., Armand P., Scott E.C., Halwani A., Gutierrez M., Millenson M.M., Cohen A.D., Schuster S.J., Lebovic D. (2016). Nivolumab in Patients With Relapsed or Refractory Hematologic Malignancy: Preliminary Results of a Phase Ib Study. J. Clin. Oncol..

[27] Ansell S.M., Minnema M.C., Johnson P., Timmerman J.M., Armand P., Shipp M.A., Rodig S.J., Ligon A.H., Roemer M.G., Reddy N. (2019). Nivolumab for Relapsed/Refractory Diffuse Large B-Cell Lymphoma in Patients Ineligible for or Having Failed Autologous Transplantation: A Single-Arm, Phase II Study. J. Clin. Oncol..

[28] Armand P., Rodig S., Melnichenko V., Thieblemont C., Bouabdallah K., Tumyan G., Özcan M., Portino S., Fogliatto L., Caballero M.D. (2019). Pembrolizumab in Relapsed or Refractory Primary Mediastinal Large B-Cell Lymphoma. J. Clin. Oncol..

[29] Ansell S., Gutierrez M.E., Shipp M.A., Gladstone D., Moskowitz A., Borello I., Popa-McKiver M., Farsaci B., Zhu M.L., Lesokhin A.M. (2016). A Phase 1 Study of Nivolumab in Combination with Ipilimumab for Relapsed or Refractory Hematologic Malignancies (CheckMate 039). Blood.

[30] Nastoupil L.J., Westin J., Fowler N., Fanale M.A., Samaniego F., Oki Y., Obi C., Cao J., Cheng X., Ma M.C.J. (2017). Response rates with pembrolizumab in combination with rituximab in patients with relapsed follicular lymphoma: Interim results of an on open-label, phase II study. J. Clin. Oncol..

[31] Westin J.R., Chu F., Zhang M., Fayad L.E., Kwak L.W., Fowler N., Romaguera J., Hagemeister F.B., Fanale M.A., Samaniego F. (2014). Safety and activity of PD1 blockade by pidilizumab in combination with rituximab in patients with relapsed follicular lymphoma: A single group, open-label, phase 2 trial. Lancet Oncol..

[32] Witzig T.E., Vukov A.M., Habermann T.M., Geyer S., Kurtin P.J., Friedenberg W.R., White W.L., Chalchal H.I., Flynn P.J., Fitch T.R. (2005). Rituximab Therapy for Patients With Newly Diagnosed, Advanced-Stage, Follicular Grade I Non-Hodgkin’s Lymphoma: A Phase II Trial in the North Central Cancer Treatment Group. J. Clin. Oncol..

[33] Hainsworth J.D., Burris H.A., Morrissey L.H., Litchy S., Scullin D.C., Bearden J.D., Richards P., Greco F.A. (2000). Rituximab monoclonal antibody as initial systemic therapy for patients with low-grade non-Hodgkin lymphoma. Blood.

[34] Davis T.A., Grillo-Lopez A.J., White C.A., McLaughlin P., Czuczman M.S., Link B.K., Maloney D.G., Weaver R.L., Rosenberg J., Levy R. (2000). Rituximab Anti-CD20 Monoclonal Antibody Therapy in Non-Hodgkin’s Lymphoma: Safety and Efficacy of Re-Treatment. J. Clin. Oncol..

[35] Palomba M., Till B., Park S., Morschhauser F., Cartron G., Marks R., Penuel E., Chitra S., Kuhn M., Popplewell L. (2017). A phase Ib study evaluating the safety and clinical activity of atezolizumab combined with obinutuzumab in patients with relapsed or refractory non-hodgkin lymphoma (NHL). Hematol. Oncol..

[36] Younes A., John B.M., Diefenbach C.S., Ferrari S., Kahn C., Sharman J.P., Tani M., Ujjani C.S., Vitolo U., Yuen S. (2017). Safety and Efficacy of Atezolizumab in Combination with Obinutuzumab and Bendamustine in Patients with Previously Untreated Follicular Lymphoma: An Interim Analysis. Blood.

[37] Younes A., Burke J.M., Cheson B., Diefenbach C., Ferrari S., Hahn U., Hawkes E., Khan C., Lossos I.S., Musuraka G. (2018). Safety and Efficacy of Atezolizumab in Combination with Rituximab Plus CHOP in Previously Untreated Patients with Diffuse Large B-Cell Lymphoma (DLBCL): Primary Analysis of a Phase I/II Study. Blood.

[38] Bond D.A., Yildiz V., Wei L., Alinari L., William B.M., Brammer J.E., Christian B.A., Blum K.A., Maddocks K.J. (2019). A Phase I Study of Nivolumab and Lenalidomide in Relapsed/ Refractory B Cell Lymphoma. Blood.

[39] Casulo C., Santoro A., Ando K., Le Gouill S., Ruan J., Radford J., Arcaini L., Pinto A., Bouabdallah R., Izutsu K. (2019). Durvalumab (Anti PD-L1) As Monotherapy or in Combination Therapy for Relapsed/Refractory (r/r) Diffuse Large B-Cell Lymphoma (DLBCL) and Follicular Lymphoma (FL): A Subgroup Analysis from the Phase 1/2 Fusion NHL-001 Global Multicenter Trial. Blood.

[40] Khouri I.F., Curbelo I.F., Bassett R.L., Allison J.P., Gulbis A.M., Seliger B., Turturro F., Jabbour E.J., Milton D.R., Vence L.M. (2017). Ipilimumab plus Lenalidomide after Allogeneic and Autologous Stem Cell Transplantation for Patients with Lymphoid Malignancies. Clin. Cancer Res..

[41] Younes A., Brody J., Carpio C., Lopez-Guillermo A., Ben-Yehuda D., Ferhanoglu B., Nagler A., Ozcan M., Avivi I., Bosch F. (2019). Safety and activity of ibrutinib in combination with nivolumab in patients with relapsed non-Hodgkin lymphoma or chronic lymphocytic leukaemia: A phase 1/2a study. Lancet Haematol..

[42] Wilson W.H., Young R.M., Schmitz R., Yang Y., Pittaluga S., Wright G., Lih C.-J., Williams P.M., Shaffer A.L., Gerecitano J. (2015). Targeting B cell receptor signaling with ibrutinib in diffuse large B cell lymphoma. Nat. Med..

[43] Bartlett N.L., Costello B.A., LaPlant B.R., Ansell S.M., Kuruvilla J.G., Reeder C.B., Thye L.S., Anderson D.M., Krysiak K., Ramirez C. (2018). Single-agent ibrutinib in relapsed or refractory follicular lymphoma: A phase 2 consortium trial. Blood.

[44] Byrd J.C., Brown J.R., O’Brien S., Barrientos J.C., Kay N.E., Reddy N.M., Coutre S., Tam C.S., Mulligan S.P., Jaeger U. (2014). Ibrutinib versus Ofatumumab in Previously Treated Chronic Lymphoid Leukemia. N. Engl. J. Med..

[45] Herrera A.F., Goy A., Mehta A., Ramchandren R., Pagel J.M., Svoboda J., Guan S., Hill J.S., Kwei K., Liu E.A. (2020). Safety and activity of ibrutinib in combination with durvalumab in patients with relapsed or refractory follicular lymphoma or diffuse large B-cell lymphoma. Am. J. Hematol..

[46] Zinzani P.L., Santoro A., Gritti G., Brice P., Barr P.M., Kuruvilla J., Cunningham D., Kline J., Johnson N.A., Mehta-Shah N. (2019). Nivolumab Combined With Brentuximab Vedotin for Relapsed/Refractory Primary Mediastinal Large B-Cell Lymphoma: Efficacy and Safety From the Phase II CheckMate 436 Study. J. Clin. Oncol..

[47] Frigault M.J., Armand P., Redd R., Jeter E., Merryman R.W., Coleman K.C., Herrera A.F., Dahi P., Nieto Y., LaCasce A. (2020). PD-1 blockade for diffuse large B-cell lymphoma after autologous stem cell transplantation. Blood Adv..

[48] Goebeler M.-E., Knop S., Viardot A., Kufer P., Topp M.S., Einsele H., Noppeney R., Hess G., Kallert S., Mackensen A. (2016). Bispecific T-Cell Engager (BiTE) Antibody Construct Blinatumomab for the Treatment of Patients With Relapsed/Refractory Non-Hodgkin Lymphoma: Final Results From a Phase I Study. J. Clin. Oncol..

[49] Viardot A., Goebeler M.-E., Hess G., Neumann S., Pfreundschuh M., Adrian N., Zettl F., Libicher M., Sayehli C., Stieglmaier J. (2016). Phase 2 study of the bispecific T-cell engager (BiTE) antibody blinatumomab in relapsed/refractory diffuse large B-cell lymphoma. Blood.

[50] Coyle L., Morley N.J., Rambaldi A., Mason K.D., Verhoef G., Furness C.L., Zhang A., Jung A.S., Cohan D., Franklin J.L. (2020). Open-Label, phase 2 study of blinatumomab as second salvage therapy in adults with relapsed/refractory aggressive B-cell non-Hodgkin lymphoma. Leuk. Lymphoma.

[51] Poh C., Frankel P., Ruel C., Abedi M., Schwab E., Costello C.L., Zain J., Budde L.E., William B.M., Foss F.M. (2019). Blinatumomab/Lenalidomide in Relapsed/Refractory Non-Hodgkin’s Lymphoma: A Phase I California Cancer Consortium Study of Safety, Efficacy and Immune Correlative Analysis. Blood.

[52] Katz D.A., Chu M.P., David K.A., Thieblemont C., Morley N.J., Khan S.S., Chen Y., Kalabus J., Morris J., Anderson A. (2019). Open-Label, Phase 2 Study of Blinatumomab after First-Line Rituximab-Chemotherapy in Adults with Newly Diagnosed, High-Risk Diffuse Large B-Cell Lymphoma. Blood.

[53] Schuster S.J., Bartlett N.L., Assouline S., Yoon S.-S., Bosch F., Sehn L.H., Cheah C.Y., Shadman M., Gregory G.P., Ku M. (2019). Mosunetuzumab Induces Complete Remissions in Poor Prognosis Non-Hodgkin Lymphoma Patients, Including Those Who Are Resistant to or Relapsing After Chimeric Antigen Receptor T-Cell (CAR-T) Therapies, and Is Active in Treatment through Multiple Lines. Blood.

[54] Hutchings M., Lugtenburg P., Mous R., Clausen M.R., Chamuleau M., Linton K., Rule S., Lopez J.S., Oliveri R.S., Demarco D. (2020). Epcoritamab (GEN3013; DuoBody-CD3×CD20) to induce complete response in patients with relapsed/refractory B-cell non-Hodgkin lymphoma (B-NHL): Complete dose escalation data and efficacy results from a phase I/II trial. J. Clin. Oncol..

[55] Bannerji R., Advani R.H., Brown J.R., Arnason J.E., Barnes J.A., Allan J.N., Ansell S.M., O’Brien S.M., Chavez J.C., Adriaens L. (2017). Safety and Preliminary Clinical Activity of REGN1979, an Anti-CD20 x Anti-CD3 Bispecific Antibody, in Patients with B-NHL Previously Treated with CD20-Directed Antibody Therapy. Blood.

[56] Bannerji M.R., Allan J.N., Arnason J.E., Brown J.R., Advani R., Ansell S.M., O’Brien S.M., Duell J., Martin F.P., Joyce R.M. (2020). Odronextamab (REGN1979), a Human CD20 x CD3 Bispecific Antibody, Induces Durable, Complete Responses in Patients with Highly Refractory B-Cell Non-Hodgkin Lymphoma, Including Patients Refractory to CAR T Therapy. Blood.

[57] Morschhauser F., Carlo-Stella C., Offner F., Salles G.A., Hutchings M., Iacoboni G., Sureda A., Crump M., Martinez-Lopez J., Thomas D. (2019). Dual CD20-Targeted Therapy With Concurrent CD20-TCB and Obinutuzumab Shows Highly Promising Clinical Activity and Manageable Safety in Relapsed or Refractory B-Cell Non-Hodgkin Lymphoma: Preliminary Results From a Phase Ib Trial. Blood.

[58] Wong R., Pepper C., Brennan P., Nagorsen D., Man S., Fegan C. (2013). Blinatumomab induces autologous T-cell killing of chronic lymphocytic leukemia cells. Haematol..

[59] Robinson H.R., Qi J., Cook E.M., Nichols C., Dadashian E.L., Underbayev C., Herman S.E.M., Saba N.S., Keyvanfar K., Sun C. (2018). A CD19/CD3 bispecific antibody for effective immunotherapy of chronic lymphocytic leukemia in the ibrutinib era. Blood.

[60] Martens A.W.J., Janssen S.R., Derks I.A.M., Iii H.C.A., Izhak L., Van Kampen R., Tonino S.H., Eldering E., Van Der Windt G.J.W., Kater A.P. (2020). CD3xCD19 DART molecule treatment induces non-apoptotic killing and is efficient against high-risk chemotherapy and venetoclax-resistant chronic lymphocytic leukemia cells. J. Immunother. Cancer.

[61] Circosta P., Elia A.R., Landra I., Machiorlatti R., Todaro M., Aliberti S., Brusa D., Deaglio S., Chiaretti S., Bruna R. (2018). Tailoring CD19xCD3-DART exposure enhances T-cells to eradication of B-cell neoplasms. OncoImmunology.

[62] Gohil S.H., Evans R., Harasser M., El-Kholy M., Paredes-Moscosso S., Della Peruta M., Nathwani A.C. (2019). Ibrutinib enhances the efficacy of ROR1 bispecific T cell engager mediated cytotoxicity in chronic lymphocytic leukaemia. Br. J. Haematol..

[63] Park J.H., Rivière I., Gonen M., Wang X., Sénéchal B., Curran K.J., Sauter C., Wang Y., Santomasso B., Mead E. (2018). Long-Term Follow-up of CD19 CAR Therapy in Acute Lymphoblastic Leukemia. N. Engl. J. Med..

[64] Schuster S.J., Bishop M.R., Tam C.S., Waller E.K., Borchmann P., McGuirk J.P., Jäger U., Jaglowski S., Andreadis C., Westin J.R. (2019). Tisagenlecleucel in Adult Relapsed or Refractory Diffuse Large B-Cell Lymphoma. N. Engl. J. Med..

[65] Wang M., Munoz J., Goy A., Locke F.L., Jacobson C.A., Hill B.T., Timmerman J.M., Holmes H., Jaglowski S., Flinn I.W. (2020). KTE-X19 CAR T-Cell Therapy in Relapsed or Refractory Mantle-Cell Lymphoma. N. Engl. J. Med..

[66] Till B.G., Jensen M.C., Wang J., Chen E.Y., Wood B.L., Greisman H.A., Qian X., James S.E., Raubitschek A., Forman S.J. (2008). Adoptive immunotherapy for indolent non-Hodgkin lymphoma and mantle cell lymphoma using genetically modified autologous CD20-specific T cells. Blood.

[67] Kochenderfer J.N., Dudley M.E., Feldman S.A., Wilson W.H., Spaner D.E., Maric I., Stetler-Stevenson M., Phan G.Q., Hughes M.S., Sherry R.M. (2012). B-cell depletion and remissions of malignancy along with cytokine-associated toxicity in a clinical trial of anti-CD19 chimeric-antigen-receptor–transduced T cells. Blood.

[68] Kochenderfer J.N., Dudley M.E., Carpenter R.O., Kassim S.H., Rose J.J., Telford W.G., Hakim F.T., Halverson D.C., Fowler D.H., Hardy N.M. (2013). Donor-derived CD19-targeted T cells cause regression of malignancy persisting after allogeneic hematopoietic stem cell transplantation. Blood.

[69] Brentjens R.J., Rivière I., Park J.H., Davila M.L., Wang X., Stefanski J., Taylor C., Yeh R., Bartido S., Borquez-Ojeda O. (2011). Safety and persistence of adoptively transferred autologous CD19-targeted T cells in patients with relapsed or chemotherapy refractory B-cell leukemias. Blood.

[70] Kochenderfer J.N., Dudley M.E., Kassim S.H., Somerville R.P., Carpenter R.O., Stetler-Stevenson M., Yang J.C., Phan G.Q., Hughes M.S., Sherry R.M. (2015). Chemotherapy-Refractory Diffuse Large B-Cell Lymphoma and Indolent B-Cell Malignancies Can Be Effectively Treated With Autologous T Cells Expressing an Anti-CD19 Chimeric Antigen Receptor. J. Clin. Oncol..

[71] Kochenderfer J.N., Somerville R.P., Lu T., Shi V., Bot A., Rossi J., Xue A., Goff S.L., Yang J.C., Sherry R.M. (2017). Lymphoma Remissions Caused by Anti-CD19 Chimeric Antigen Receptor T Cells Are Associated With High Serum Interleukin-15 Levels. J. Clin. Oncol..

[72] Ramos C.A., Savoldo B., Torrano V., Ballard B., Zhang H., Dakhova O., Liu E., Carrum G., Kamble R.T., Gee A.P. (2016). Clinical responses with T lymphocytes targeting malignancy-associated κ light chains. J. Clin. Investig..

[73] Geyer M.B., Rivière I., Sénéchal B., Wang X., Wang Y., Purdon T.J., Hsu M., Devlin S.M., Halton E., Lamanna N. (2018). Autologous CD19-Targeted CAR T Cells in Patients with Residual CLL following Initial Purine Analog-Based Therapy. Mol. Ther..

[74] Locke F.L., Ghobadi A., Jacobson C.A., Miklos D.B., Lekakis L.J., Oluwole O., Lin Y., Braunschweig I., Hill B.T., Timmerman J.M. (2019). Long-term safety and activity of axicabtagene ciloleucel in refractory large B-cell lymphoma (ZUMA-1): A single-arm, multicentre, phase 1–2 trial. Lancet Oncol..

[75] Jacobson C.A., Chavez J.C., Sehgal A.R., William B.M., Munoz J., Salles G.A., Casulo C., Munshi P.N., Maloney D.G., De Vos S. (2020). Interim analysis of ZUMA-5: A phase II study of axicabtagene ciloleucel (axi-cel) in patients (pts) with relapsed/refractory indolent non-Hodgkin lymphoma (R/R iNHL). J. Clin. Oncol..

[76] Fraietta J.A., Lacey S.F., Orlando E.J., Pruteanu-Malinici I., Gohil M., Lundh S., Boesteanu A.C., Wang Y., O’Connor R.S., Hwang W.-T. (2018). Determinants of response and resistance to CD19 chimeric antigen receptor (CAR) T cell therapy of chronic lymphocytic leukemia. Nat. Med..

[77] Hirayama A.V., Gauthier J., Hay K.A., Voutsinas J.M., Wu Q., Pender B.S., Hawkins R.M., Vakil A., Steinmetz R.N., Riddell S.R. (2019). High rate of durable complete remission in follicular lymphoma after CD19 CAR-T cell immunotherapy. Blood.

[78] Porter D.L., Hwang W.-T., Frey N.V., Lacey S.F., Shaw P.A., Loren A.W., Bagg A., Marcucci K.T., Shen A., Gonzalez V. (2015). Chimeric antigen receptor T cells persist and induce sustained remissions in relapsed refractory chronic lymphocytic leukemia. Sci. Transl. Med..

[79] Schuster S.J., Svoboda J., Chong E.A., Nasta S.D., Mato A., Anak Ö., Brogdon J.L., Pruteanu-Malinici I., Bhoj V., Landsburg D. (2017). Chimeric Antigen Receptor T Cells in Refractory B-Cell Lymphomas. N. Engl. J. Med..

[80] Turtle C.J., Hay K.A., Hanafi L.-A., Li D., Cherian S., Chen X., Wood B., Lozanski A., Byrd J.C., Heimfeld S. (2017). Durable Molecular Remissions in Chronic Lymphocytic Leukemia Treated With CD19-Specific Chimeric Antigen Receptor–Modified T Cells After Failure of Ibrutinib. J. Clin. Oncol..

[81] Abramson M.J.S., Palomba M.L., Gordon L.I., Lunning D.M.A., Wang M.L., Arnason J.E., Mehta A., Purev E., Maloney D.G., Andreadis M.C. (2019). Pivotal Safety and Efficacy Results from Transcend NHL 001, a Multicenter Phase 1 Study of Lisocabtagene Maraleucel (liso-cel) in Relapsed/Refractory (R/R) Large B Cell Lymphomas. Blood.

[82] Ying Z., He T., Wang X., Zheng W., Lin N., Tu M., Xie Y., Ping L., Zhang C., Liu W. (2019). Parallel Comparison of 4-1BB or CD28 Co-stimulated CD19-Targeted CAR-T Cells for B Cell Non-Hodgkin’s Lymphoma. Mol. Ther. Oncol..

[83] Siddiqi T., Soumerai J.D., Dorritie K.A., Stephens D.M., Riedell P.A., Arnason J.E., Kipps T.J., Gillenwater H.H., Gong L., Dubovsky J.A. (2019). Rapid Undetectable MRD (uMRD) Responses in Patients with Relapsed/Refractory (R/R) Chronic Lymphocytic Leukemia/Small Lymphocytic Lymphoma (CLL/SLL) Treated with Lisocabtagene Maraleucel (liso-cel), a CD19-Directed CAR T Cell Product: Updated Results from Transcend CLL 004, a Phase 1/2 Study Including Patients with High-Risk Disease Previously Treated with Ibrutinib. Blood.

[84] Fraietta J.A., Beckwith K.A., Patel P.R., Ruella M., Zheng Z., Barrett D.M., Lacey S.F., Melenhorst J.J., Mcgettigan S.E., Cook D.R. (2016). Ibrutinib enhances chimeric antigen receptor T-cell engraftment and efficacy in leukemia. Blood.

[85] Cao Y., Lu W., Sun R., Jin X., Cheng L., He X., Wang L., Yuan T., Lyu C., Zhao M. (2019). Anti-CD19 Chimeric Antigen Receptor T Cells in Combination With Nivolumab Are Safe and Effective Against Relapsed/Refractory B-Cell Non-hodgkin Lymphoma. Front. Oncol..

[86] Wang X., Popplewell L.L., Wagner J.R., Naranjo A., Blanchard M.S., Mott M.R., Norris A.P., Wong C.W., Urak R.Z., Chang W.-C. (2016). Phase 1 studies of central memory–derived CD19 CAR T–cell therapy following autologous HSCT in patients with B-cell NHL. Blood.

[87] Kalos M., Levine B.L., Porter D.L., Katz S., Grupp S.A., Bagg A., June C.H. (2011). T Cells with Chimeric Antigen Receptors Have Potent Antitumor Effects and Can Establish Memory in Patients with Advanced Leukemia. Sci. Transl. Med..

[88] Gauthier J., Hirayama A.V., Purushe J., Hay K.A., Lymp J., Li D.H., Yeung C.C.S., Sheih A., Pender B.S., Hawkins R.M. (2020). Feasibility and efficacy of CD19-targeted CAR T cells with concurrent ibrutinib for CLL after ibrutinib failure. Blood.

[89] Alexandrov L.B., Nik-Zainal S., Wedge D.C., Aparicio S.A.J.R., Behjati S., Biankin A.V., Bignell G.R., Bolli N., Borg A., Børresen-Dale A.-L. (2013). Signatures of mutational processes in human cancer. Nature.

[90] Samstein R.M., Lee C.-H., Shoushtari A.N., Hellmann M.D., Shen R., Janjigian Y.Y., Barron D.A., Zehir A., Jordan E.J., Omuro A. (2019). Tumor mutational load predicts survival after immunotherapy across multiple cancer types. Nat. Genet..

[91] Rajasagi M., Shukla S.A., Fritsch E.F., Keskin D.B., DeLuca D., Carmona E., Zhang W., Sougnez C., Cibulskis K., Sidney J. (2014). Systematic identification of personal tumor-specific neoantigens in chronic lymphocytic leukemia. Blood.

[92] Hu Z., Anandappa A.J., Sun J., Kim J., Leet D.E., Bozym D.J., Chen C., Williams L., Shukla S.A., Zhang W. (2018). A cloning and expression system to probe T-cell receptor specificity and assess functional avidity to neoantigens. Blood.

[93] Khodadoust M.S., Olsson N., Wagar L.E., Haabeth O.A.W., Chen B., Swaminathan K., Rawson K., Liu C.L., Steiner D., Lund P. (2017). Antigen presentation profiling reveals recognition of lymphoma immunoglobulin neoantigens. Nat. Cell Biol..

[94] Martin M.D., Badovinac V.P. (2018). Defining Memory CD8 T Cell. Front. Immunol..

[95] Sathaliyawala T., Kubota M., Yudanin N., Turner D., Camp P., Thome J.J.C., Bickham K.L., Lerner H., Goldstein M., Sykes M. (2013). Distribution and Compartmentalization of Human Circulating and Tissue-Resident Memory T Cell Subsets. Immunity.

[96] Mahnke Y.D., Brodie T., Sallusto F., Roederer M., Lugli E. (2013). The who’s who of T-cell differentiation: Human memory T-cell subsets. Eur. J. Immunol..

[97] Pourgheysari B., Bruton R., Parry H., Billingham L., Fegan C., Murray J., Moss P. (2010). The number of cytomegalovirus-specific CD4+ T cells is markedly expanded in patients with B-cell chronic lymphocytic leukemia and determines the total CD4+ T-cell repertoire. Blood.

[98] D’Arena G., Laurenti L., Minervini M.M., Deaglio S., Bonello L., De Martino L., De Padua L., Savino L., Tarnani M., De Feo V. (2011). Regulatory T-cell number is increased in chronic lymphocytic leukemia patients and correlates with progressive disease. Leuk. Res..

[99] Tonino S.H., Van De Berg P.J., La Yong S., Berge I.J.T., Kersten M.J., Van Lier R.A.W., Van Oers M.H., Kater A.P. (2012). Expansion of effector T cells associated with decreased PD-1 expression in patients with indolent B cell lymphomas and chronic lymphocytic leukemia. Leuk. Lymphoma.

[100] Mackus W.J.M., Frakking F.N.J., Grummels A., Gamadia L.E., De Bree G.J., Hamann D., Van Lier R.A., Van Oers M.H.J. (2003). Expansion of CMV-specific CD8+CD45RA+CD27- T cells in B-cell chronic lymphocytic leukemia. Blood.

[101] Nunes C., Wong R., Mason M., Fegan C., Man S., Pepper C. (2011). Expansion of a CD8+PD-1+ Replicative Senescence Phenotype in Early Stage CLL Patients Is Associated with Inverted CD4:CD8 Ratios and Disease Progression. Clin. Cancer Res..

[102] Gonzalez-Rodriguez A.P., Contesti J., Huergo-Zapico L., Lopez-Larrea C., Fernández-Guizán A., Acebes-Huerta A., Gonzalez-Huerta A.J., Gonzalez E., Fernandez-Alvarez C., Gonzalez S. (2010). Prognostic significance of CD8 and CD4 T cells in chronic lymphocytic leukemia. Leuk. Lymphoma.

[103] Palma M., Gentilcore G., Heimersson K., Mozaffari F., Näsman-Glaser B., Young E., Rosenquist R., Hansson L., Österborg A., Mellstedt H. (2017). T cells in chronic lymphocytic leukemia display dysregulated expression of immune checkpoints and activation markers. Haematologica.

[104] De Weerdt I., Hofland T., De Boer R., Dobber J.A., Dubois J., Van Nieuwenhuize D., Mobasher M., De Boer F., Hoogendoorn M., Velders G.A. (2019). Distinct immune composition in lymph node and peripheral blood of CLL patients is reshaped during venetoclax treatment. Blood Adv..

[105] Christopoulos P., Pfeifer D., Bartholomé K., Follo M., Timmer J., Fisch P., Veelken H. (2011). Definition and characterization of the systemic T-cell dysregulation in untreated indolent B-cell lymphoma and very early CLL. Blood.

[106] Rossmann E.D., Lewin N., Jeddi-Tehrani M., Österborg A., Mellstedt H. (2002). Intracellular T cell cytokines in patients with B cell chronic lymphocytic leukaemia (B-CLL). Eur. J. Haematol..

[107] Görgün G., Holderried T.A.W., Zahrieh D., Neuberg D., Gribben J.G. (2005). Chronic lymphocytic leukemia cells induce changes in gene expression of CD4 and CD8 T cells. J. Clin. Investig..

[108] Podhorecka M., Dmoszynska A., Rolinski J., Wasik E. (2002). T type 1/type 2 subsets balance in B-cell chronic lymphocytic leukemia—The three-color flow cytometry analysis. Leuk. Res..

[109] Pangault C., Amé-Thomas P., Ruminy P., Rossille D., Caron G., Baia M., De Vos J., Roussel M., Monvoisin C., Lamy T. (2010). Follicular lymphoma cell niche: Identification of a preeminent IL-4-dependent TFH–B cell axis. Leukemia.

[110] Hilchey S.P., Rosenberg A.F., Hyrien O., Secor-Socha S., Cochran M., Brady M.T., Wang J.-C.E., Sanz I., Burack W.R., Quataert S.A. (2011). Follicular lymphoma tumor–infiltrating T-helper (TH) cells have the same polyfunctional potential as normal nodal TH cells despite skewed differentiation. Blood.

[111] Glas A.M., Knoops L., Delahaye L., Kersten M.J., Kibbelaar R.E., Wessels L.A., Van Laar R., Van Krieken J.H.J.M., Baars J.W., Raemaekers J. (2007). Gene-Expression and Immunohistochemical Study of Specific T-Cell Subsets and Accessory Cell Types in the Transformation and Prognosis of Follicular Lymphoma. J. Clin. Oncol..

[112] Edinger J.T., Kant J.A., Swerdlow S.H. (2010). Cutaneous Marginal Zone Lymphomas Have Distinctive Features and Include 2 Subsets. Am. J. Surg. Pathol..

[113] Koulis A., Diss T., Isaacson P.G., Dogan A. (1997). Characterization of tumor-infiltrating T lymphocytes in B-cell lymphomas of mucosa-associated lymphoid tissue. Am. J. Pathol..

[114] Riedel M.K.S. (2001). CD4 + Th1-cells Predominate in Low-grade B-Cell Lymphoma of Gastric Mucosa-associated Lymphoid Tissue (MALT type). Scand. J. Gastroenterol..

[115] Vincent-Fabert C., Soubeyran I., Velasco V., Parrens M., Jeannet R., Lereclus E., Gachard N., Feuillard J., Faumont N. (2019). Inflamed phenotype of splenic marginal zone B-cell lymphomas with expression of PD-L1 by intratumoral monocytes/macrophages and dendritic cells. Cell. Mol. Immunol..

[116] Andorsky D.J., Yamada R.E., Said J., Pinkus G.S., Betting D.J., Timmerman J.M. (2011). Programmed Death Ligand 1 Is Expressed by Non-Hodgkin Lymphomas and Inhibits the Activity of Tumor-Associated T Cells. Clin. Cancer Res..

[117] Sommermeyer D., Hudecek M., Kosasih P.L., Gogishvili T., Maloney D.G., Turtle C.J., Riddell S.R. (2016). Chimeric antigen receptor-modified T cells derived from defined CD8+ and CD4+ subsets confer superior antitumor reactivity in vivo. Leukemia.

[118] Catakovic K., Klieser E., Neureiter D., Geisberger R. (2017). T cell exhaustion: From pathophysiological basics to tumor immunotherapy. Cell Commun. Signal..

[119] Li L., Zhang J., Chen J., Xu-Monette Z.Y., Miao Y., Xiao M., Young K.H., Wang S., Medeiros L.J., Wang M. (2018). B-cell receptor–mediated NFATc1 activation induces IL-10/STAT3/PD-L1 signaling in diffuse large B-cell lymphoma. Blood.

[120] Kiyasu J., Miyoshi H., Hirata A., Arakawa F., Ichikawa A., Niino D., Sugita Y., Yufu Y., Choi I., Abe Y. (2015). Expression of programmed cell death ligand 1 is associated with poor overall survival in patients with diffuse large B-cell lymphoma. Blood.

[121] Kwon D., Kim S., Kim P.-J., Go H., Nam S.J., Paik J.H., Kim Y.A., Kim T.M., Heo D.S., Kim C.W. (2015). Clinicopathological analysis of programmed cell death 1 and programmed cell death ligand 1 expression in the tumour microenvironments of diffuse large B cell lymphomas. Histopathology.

[122] Chen B.J., Dashnamoorthy R., Galera P., Makarenko V., Chang H., Ghosh S., Evens A.M. (2019). The immune checkpoint molecules PD-1, PD-L1, TIM-3 and LAG-3 in diffuse large B-cell lymphoma. Oncotarget.

[123] Godfrey J., Tumuluru S., Bao R., Leukam M., Venkataraman G., Phillip J., Fitzpatrick C., McElherne J., MacNabb B.W., Orlowski R. (2019). PD-L1 gene alterations identify a subset of diffuse large B-cell lymphoma harboring a T-cell–inflamed phenotype. Blood.

[124] Zhang L., Du H., Xiao T., Liu J.-Z., Liu G., Wang J.-X., Li G.-Y., Wang L.-X. (2015). Prognostic value of PD-1 and TIM-3 on CD3+ T cells from diffuse large B-cell lymphoma. Biomed. Pharmacother..

[125] Li L., Sun R., Miao Y., Tran T., Adams L., Roscoe N., Xu B., Manyam G.C., Tan X., Zhang H. (2019). PD-1/PD-L1 expression and interaction by automated quantitative immunofluorescent analysis show adverse prognostic impact in patients with diffuse large B-cell lymphoma having T-cell infiltration: A study from the International DLBCL Consortium Program. Mod. Pathol..

[126] Xiao T., Zhang L., Chen L., Liu G., Feng Z., Gao L. (2014). Tim-3 expression is increased on peripheral T cells from diffuse large B cell lymphoma. Tumor Biol..

[127] Josefsson S.E., Beiske K., Blaker Y.N., Førsund M.S., Holte H., Østenstad B., Kimby E., Köksal H., Wälchli S., Bai B. (2019). TIGIT and PD-1 Mark Intratumoral T Cells with Reduced Effector Function in B-cell Non-Hodgkin Lymphoma. Cancer Immunol. Res..

[128] Laurent C., Charmpi K., Gravelle P., Tosolini M., Franchet C., Ysebaert L., Brousset P., Bidaut A., Ycart B., Fournié J.-J. (2015). Several immune escape patterns in non-Hodgkin’s lymphomas. OncoImmunology.

[129] Xu-Monette Z.Y., Xiao M., Au Q., Padmanabhan R., Xu B., Hoe N., Rodríguez-Perales S., Torres-Ruiz R., Manyam G.C., Visco C. (2019). Immune Profiling and Quantitative Analysis Decipher the Clinical Role of Immune-Checkpoint Expression in the Tumor Immune Microenvironment of DLBCL. Cancer Immunol. Res..

[130] Motta M., Rassenti L., Shelvin B.J., Lerner S., Kipps T.J., Keating M.J., Wierda W.G. (2005). Increased expression of CD152 (CTLA-4) by normal T lymphocytes in untreated patients with B-cell chronic lymphocytic leukemia. Leukemia.

[131] Ramsay A.G., Clear A.J., Fatah R., Gribben J.G. (2012). Multiple inhibitory ligands induce impaired T-cell immunologic synapse function in chronic lymphocytic leukemia that can be blocked with lenalidomide: Establishing a reversible immune evasion mechanism in human cancer. Blood.

[132] Riches J.C., Davies J.K., McClanahan F., Fatah R., Iqbal S., Agrawal S., Ramsay A.G., Gribben J.G. (2013). T cells from CLL patients exhibit features of T-cell exhaustion but retain capacity for cytokine production. Blood.

[133] Catakovic K., Gassner F.J., Ratswohl C., Zaborsky N., Rebhandl S., Schubert M., Steiner M., Gutjahr J.C., Pleyer L., Egle A. (2017). TIGIT expressing CD4+T cells represent a tumor-supportive T cell subset in chronic lymphocytic leukemia. OncoImmunology.

[134] Yang Z.-Z., Grote D.M., Ziesmer S.C., Niki T., Hirashima M., Novak A.J., Witzig T.E., Ansell S.M. (2012). IL-12 upregulates TIM-3 expression and induces T cell exhaustion in patients with follicular B cell non-Hodgkin lymphoma. J. Clin. Investig..

[135] Yang Z.-Z., Grote D.M., Ziesmer S.C., Xiu B., Novak A.J., Ansell S.M. (2015). PD-1 expression defines two distinct T-cell sub-populations in follicular lymphoma that differentially impact patient survival. Blood Cancer J..

[136] Gravelle P., Do C., Franchet C., Mueller S., Oberic L., Ysebaert L., LaRocca L.M., Hohaus S., Calmels M.-N., Frenois F.-X. (2016). Impaired functional responses in follicular lymphoma CD8+TIM-3+ T lymphocytes following TCR engagement. OncoImmunology.

[137] Yang Z.-Z., Kim H.J., Villasboas J.C., Chen Y.-P., Price-Troska T., Jalali S., Wilson M., Novak A.J., Ansell S.M. (2017). Expression of LAG-3 defines exhaustion of intratumoral PD-1+ T cells and correlates with poor outcome in follicular lymphoma. Oncotarget.

[138] Josefsson S.E., Huse K., Kolstad A., Beiske K., Pende D., Steen C.B., Inderberg E.M., Lingjærde O.C., Østenstad B., Smeland E.B. (2018). T Cells Expressing Checkpoint Receptor TIGIT Are Enriched in Follicular Lymphoma Tumors and Characterized by Reversible Suppression of T-cell Receptor Signaling. Clin. Cancer Res..

[139] Myklebust J.H., Irish J.M., Brody J., Czerwinski D.K., Houot R., Kohrt H.E., Timmerman J., Said J., Green M.R., Delabie J. (2013). High PD-1 expression and suppressed cytokine signaling distinguish T cells infiltrating follicular lymphoma tumors from peripheral T cells. Blood.

[140] Hilchey S.P., De A., Rimsza L.M., Bankert R.B., Bernstein S.H. (2007). Follicular Lymphoma Intratumoral CD4+CD25+GITR+Regulatory T Cells Potently Suppress CD3/CD28-Costimulated Autologous and Allogeneic CD8+CD25−and CD4+CD25−T Cells. J. Immunol..

[141] Xerri L., Chetaille B., Serriari N., Attias C., Guillaume Y., Arnoulet C., Olive D. (2008). Programmed death 1 is a marker of angioimmunoblastic T-cell lymphoma and B-cell small lymphocytic lymphoma/chronic lymphocytic leukemia. Hum. Pathol..

[142] Harrington B.K., Wheeler E., Hornbuckle K., Shana’Ah A.Y., Youssef Y., Smith L., Ii Q.H., Klamer B., Zhang X., Long M. (2019). Modulation of immune checkpoint molecule expression in mantle cell lymphoma. Leuk. Lymphoma.

[143] Wang L., Qian J., Lu Y., Li H., Bao H., He D., Liu Z., Zheng Y., He J., Li Y. (2013). Immune evasion of mantle cell lymphoma: Expression of B7-H1 leads to inhibited T-cell response to and killing of tumor cells. Haematol..

[144] Vranic S., Ghosh N., Kimbrough J., Bilalovic N., Bender R., Arguello D., Veloso Y., Dizdarevic A., Gatalica Z. (2016). PD-L1 Status in Refractory Lymphomas. PLoS ONE.

[145] Goyal A., Moore J.B., Gimbel D., Carter J.B., Kroshinsky D., Ferry J.A., Harris N.L., Duncan L.M. (2014). PD-1, S-100 and CD1a expression in pseudolymphomatous folliculitis, primary cutaneous marginal zone B-cell lymphoma (MALT lymphoma) and cutaneous lymphoid hyperplasia. J. Cutan. Pathol..

[146] Muenst S., Hoeller S., Willi N., Dirnhofer S., Tzankov A. (2010). Diagnostic and Prognostic Utility of PD-1 In B Cell Lymphomas. Dis. Markers.

[147] Roemer M.G., Advani R.H., Ligon A.H., Natkunam Y., Redd R.A., Homer H., Connelly C.F., Sun H.H., Daadi S.E., Freeman G.J. (2016). PD-L1 and PD-L2 Genetic Alterations Define Classical Hodgkin Lymphoma and Predict Outcome. J. Clin. Oncol..

[148] Green M.R., Monti S., Rodig S.J., Juszczynski P., Currie T., O’Donnell E., Chapuy B., Takeyama K., Neuberg D., Golub T.R. (2010). Integrative analysis reveals selective 9p24.1 amplification, increased PD-1 ligand expression, and further induction via JAK2 in nodular sclerosing Hodgkin lymphoma and primary mediastinal large B-cell lymphoma. Blood.

[149] Twa D.D.W., Chan F.C., Ben-Neriah S., Woolcock B.W., Mottok A., Tan K.L., Slack G.W., Gunawardana J., Lim R.S., McPherson A.W. (2014). Genomic rearrangements involving programmed death ligands are recurrent in primary mediastinal large B-cell lymphoma. Blood.

[150] Xiong W., Chen Y., Kang X., Chen Z., Zheng P., Hsu Y.-H., Jang J.H., Qin L., Liu H., Dotti G. (2018). Immunological Synapse Predicts Effectiveness of Chimeric Antigen Receptor Cells. Mol. Ther..

[151] Buck M.D., O’Sullivan D., Pearce E.L. (2015). T cell metabolism drives immunity. J. Exp. Med..

[152] Gerriets V.A., Rathmell J.C. (2012). Metabolic pathways in T cell fate and function. Trends Immunol..

[153] Ramsay A.G., Clear A.J., Kelly G., Fatah R., Matthews J., MacDougall F., Lister T.A., Lee A.M., Calaminici M., Gribben J.G. (2009). Follicular lymphoma cells induce T-cell immunologic synapse dysfunction that can be repaired with lenalidomide: Implications for the tumor microenvironment and immunotherapy. Blood.

[154] Kiaii S., Clear A.J., Ramsay A.G., Davies D., Sangaralingam A., Lee A., Calaminici M., Neuberg D.S., Gribben J.G. (2013). Follicular Lymphoma Cells Induce Changes in T-Cell Gene Expression and Function: Potential Impact on Survival and Risk of Transformation. J. Clin. Oncol..

[155] Laurent C., Müller S., Do C., Al-Saati T., Allart S., LaRocca L.M., Hohaus S., Duchez S., Quillet-Mary A., Laurent G. (2011). Distribution, function, and prognostic value of cytotoxic T lymphocytes in follicular lymphoma: A 3-D tissue-imaging study. Blood.

[156] D’Elios M.M., Amedei A., Manghetti M., Costa F., Baldari C.T., Quazi A.S., Telford J.L., Romagnani S., Del Prete G. (1999). Impaired T-cell regulation of B-cell growth in Helicobacter pylori–related gastric low-grade MALT lymphoma. Gastroenterol..

[157] Sugiura A., Rathmell J.C. (2018). Metabolic Barriers to T Cell Function in Tumors. J. Immunol..

[158] Ricci J.-E., Chiche J. (2018). Metabolic Reprogramming of Non-Hodgkin’s B-Cell Lymphomas and Potential Therapeutic Strategies. Front. Oncol..

[159] Tosolini M., Algans C., Pont F., Ycart B., Fournié J.-J. (2016). Large-scale microarray profiling reveals four stages of immune escape in non-Hodgkin lymphomas. OncoImmunology.

[160] Siska P., Van Der Windt G.J.W., Kishton R.J., Cohen S., Eisner W., Maciver N.J., Kater A.P., Weinberg J.B., Rathmell J.C. (2016). Suppression of Glut1 and Glucose Metabolism by Decreased Akt/mTORC1 Signaling Drives T Cell Impairment in B Cell Leukemia. J. Immunol..

[161] Van Bruggen J.A.C., Martens A.W.J., Fraietta J.A., Hofland T., Tonino S.H., Eldering E., Levin M.-D., Siska P., Endstra S., Rathmell J.C. (2019). Chronic lymphocytic leukemia cells impair mitochondrial fitness in CD8+ T cells and impede CAR T-cell efficacy. Blood.

[162] Togashi Y., Shitara K., Nishikawa H. (2019). Regulatory T cells in cancer immunosuppression—Implications for anticancer therapy. Nat. Rev. Clin. Oncol..

[163] Nakayama S., Yokote T., Akioka T., Hiraoka N., Nishiwaki U., Miyoshi T., Iwaki K., Takayama A., Masuda Y., Hatooka J. (2017). Infiltration of effector regulatory T cells predicts poor prognosis of diffuse large B-cell lymphoma, not otherwise specified. Blood Adv..

[164] El-Dien M.M.S., Abdou A.G., Asaad N.Y., El-Wahed M.M.A., Kora M.A.E.-H.M. (2017). Intratumoral FOXP3+ Regulatory T Cells in Diffuse Large B-Cell Lymphoma. Appl. Immunohistochem. Mol. Morphol..

[165] Cha Z., Gu H., Zang Y., Wang Z., Li J., Huang W., Qin A., Zhu L., Tu X., Cheng N. (2018). The prevalence and function of CD4 + CXCR5 + Foxp3 + follicular regulatory T cells in diffuse large B cell lymphoma. Int. Immunopharmacol..

[166] Yang Z.-Z., Novak A.J., Stenson M.J., Witzig T.E., Ansell S.M. (2006). Intratumoral CD4+CD25+ regulatory T-cell-mediated suppression of infiltrating CD4+ T cells in B-cell non-Hodgkin lymphoma. Blood.

[167] Tzankov A., Meier C., Hirschmann P., Went P., Pileri S.A., Dirnhofer S. (2008). Correlation of high numbers of intratumoral FOXP3+ regulatory T cells with improved survival in germinal center-like diffuse large B-cell lymphoma, follicular lymphoma and classical Hodgkin’s lymphoma. Haematol..

[168] Lee A.M., Clear A.J., Calaminici M., Davies A.J., Jordan S., MacDougall F., Matthews J., Norton A.J., Gribben J.G., Lister T.A. (2006). Number of CD4+ Cells and Location of Forkhead Box Protein P3–Positive Cells in Diagnostic Follicular Lymphoma Tissue Microarrays Correlates With Outcome. J. Clin. Oncol..

[169] Carreras J., Lopez-Guillermo A., Fox B.C., Colomo L., Martinez A., Roncador G., Montserrat E., Campo E., Banham A.H. (2006). High numbers of tumor-infiltrating FOXP3-positive regulatory T cells are associated with improved overall survival in follicular lymphoma. Blood.

[170] Wahlin B.E., Aggarwal M., Montes-Moreno S., Gonzalez L.F., Roncador G., Sanchez-Verde L., Christensson B., Sander B., Kimby E. (2010). A Unifying Microenvironment Model in Follicular Lymphoma: Outcome Is Predicted by Programmed Death-1-Positive, Regulatory, Cytotoxic, and Helper T Cells and Macrophages. Clin. Cancer Res..

[171] Lim H.W., Hillsamer P., Banham A.H., Kim C.H., Banham A.H. (2005). Cutting Edge: Direct Suppression of B Cells by CD4+CD25+ Regulatory T Cells. J. Immunol..

[172] Beyer M., Kochanek M., Darabi K., Popov A., Jensen M., Endl E., Knolle P.A., Thomas R.K., Von Bergwelt-Baildon M., Debey S. (2005). Reduced frequencies and suppressive function of CD4+CD25hi regulatory T cells in patients with chronic lymphocytic leukemia after therapy with fludarabine. Blood.

[173] Jak M., Mous R., Remmerswaal E.B.M., Spijker R., Jaspers A., Yagüe A., Eldering E., Van Lier R.A.W., Van Oers M.H.J. (2009). Enhanced formation and survival of CD4+CD25hiFoxp3+T-cells in chronic lymphocytic leukemia. Leuk. Lymphoma.

[174] Piper K.P., Karanth M., McLarnon A., Kalk E., Khan N., Murray J., Pratt G., Moss P.A.H. (2011). Chronic lymphocytic leukaemia cells drive the global CD4+ T cell repertoire towards a regulatory phenotype and leads to the accumulation of CD4+ forkhead box P3+ T cells. Clin. Exp. Immunol..

[175] D’Arena G., D’Auria F., Simeon V., Laurenti L., Deaglio S., Mansueto G., Del Principe M.I., Statuto T., Pietrantuono G., Guariglia R. (2012). A shorter time to the first treatment may be predicted by the absolute number of regulatory T-cells in patients with Rai stage 0 chronic lymphocytic leukemia. Am. J. Hematol..

[176] Craig V.J., Cogliatti S.B., Arnold I., Gerke C., Balandat J.-E., Wundisch T., Müller A. (2010). B-cell receptor signaling and CD40 ligand-independent T cell help cooperate in Helicobacter-induced MALT lymphomagenesis. Leukemia.

[177] Wickenden K., Nawaz N., Mamand S., Kotecha D., Wilson A.L., Wagner S.D., Ahearne M.J. (2018). PD1hi cells associate with clusters of proliferating B-cells in marginal zone lymphoma. Diagn. Pathol..

[178] Cha Z., Qian G., Zang Y., Gu H., Huang Y., Zhu L., Li J., Liu Y., Tu X., Song H. (2017). Circulating CXCR5+CD4+ T cells assist in the survival and growth of primary diffuse large B cell lymphoma cells through interleukin 10 pathway. Exp. Cell Res..

[179] Taga K., Tosato G. (1992). IL-10 inhibits human T cell proliferation and IL-2 production. J. Immunol..

[180] Miles B., Connick E. (2018). Control of the Germinal Center by Follicular Regulatory T Cells During Infection. Front. Immunol..

[181] Pascutti M.F., Jak M., Tromp J.M., Derks I.A.M., Remmerswaal E.B.M., Thijssen R., Van Attekum M.H.A., Van Bochove G.G., Luijks D.M., Pals S.T. (2013). IL-21 and CD40L signals from autologous T cells can induce antigen-independent proliferation of CLL cells. Blood.

[182] Amé-Thomas P., Le Priol J., Yssel H., Caron G., Pangault C., Jean R., Martin N., Marafioti T., Gaulard P., Lamy T. (2011). Characterization of intratumoral follicular helper T cells in follicular lymphoma: Role in the survival of malignant B cells. Leukemia.

[183] Rawal S., Chu F., Zhang M., Park H.J., Nattamai D., Kannan S., Sharma R., Delgado D., Chou T., Lin H.Y. (2013). Cross Talk between Follicular Th Cells and Tumor Cells in Human Follicular Lymphoma Promotes Immune Evasion in the Tumor Microenvironment. J. Immunol..

[184] Amé-Thomas P., Hoeller S., Artchounin C., Misiak J., Braza M.S., Jean R., Le Priol J., Monvoisin C., Martin N., Gaulard P. (2015). CD10 delineates a subset of human IL-4 producing follicular helper T cells involved in the survival of follicular lymphoma B cells. Blood.

[185] Lu G., Middleton R.E., Sun H., Naniong M., Ott C.J., Mitsiades C.S., Wong K.-K., Bradner J.E., Jr W.G.K. (2014). The Myeloma Drug Lenalidomide Promotes the Cereblon-Dependent Destruction of Ikaros Proteins. Science.

[186] Gandhi A.K., Kang J., Havens C.G., Conklin T., Ning Y., Wu L., Ito T., Ando H., Waldman M.F., Thakurta A. (2014). Immunomodulatory agents lenalidomide and pomalidomide co-stimulate T cells by inducing degradation of T cell repressors I karos and A iolos via modulation of the E 3 ubiquitin ligase complex CRL 4 CRBN. Br. J. Haematol..

[187] Dredge K., Marriott J.B., Todryk S.M., Muller G.W., Chen R., Stirling D.I., Dalgleish A.G. (2002). Protective Antitumor Immunity Induced by a Costimulatory Thalidomide Analog in Conjunction with Whole Tumor Cell Vaccination Is Mediated by Increased Th1-Type Immunity. J. Immunol..

[188] Galustian C., Meyer B., Labarthe M.-C., Dredge K., Klaschka D., Henry J., Todryk S., Chen R., Muller G., Stirling D. (2008). The anti-cancer agents lenalidomide and pomalidomide inhibit the proliferation and function of T regulatory cells. Cancer Immunol. Immunother..

[189] Chellappa S., Kushekhar K., Munthe L.A., Tjønnfjord G.E., Aandahl E.M., Okkenhaug K., Taskén K. (2019). The PI3K p110δ Isoform Inhibitor Idelalisib Preferentially Inhibits Human Regulatory T Cell Function. J. Immunol..

[190] Chiu H., Trisal P., Bjorklund C., Carrancio S., Toraño E.G., Guarinos C., Papazoglou D., Hagner P.R., Beldi-Ferchiou A., Tarte K. (2019). Combination lenalidomide-rituximab immunotherapy activates anti-tumour immunity and induces tumour cell death by complementary mechanisms of action in follicular lymphoma. Br. J. Haematol..

[191] Ioannou N., Hagner P.R., Stokes M., Gandhi A.K., Apollonio B., Fanous M., Papazoglou D., Sutton L.A., Rosenquist R., Amini R.-M. (2020). Triggering interferon signaling in T cells with avadomide sensitizes CLL to anti-PD-L1/PD-1 immunotherapy. Blood.

[192] Görgün G., Samur M.K., Cowens K.B., Paula S., Bianchi G., Anderson J.E., White R.E., Singh A., Ohguchi H., Suzuki R. (2015). Lenalidomide Enhances Immune Checkpoint Blockade-Induced Immune Response in Multiple Myeloma. Clin. Cancer Res..

[193] Moreno L., Zabaleta A., Alignani D., Lasa M., Maiso P., Jelinek T., Segura V., Delgado J.A., Rodriguez-Otero P., Prosper F. (2016). New Insights into the Mechanism of Action (MoA) of First-in-Class IgG-Based Bcma T-Cell Bispecific Antibody (TCB) for the Treatment of Multiple Myeloma (MM). Blood.

[194] Hofland T., De Weerdt I., Ter Burg H., De Boer R., Tannheimer S., Tonino S.H., Kater A.P., Eldering E. (2019). Dissection of the Effects of JAK and BTK Inhibitors on the Functionality of Healthy and Malignant Lymphocytes. J. Immunol..

[195] Hanna B.S., Roessner P.M., Scheffold A., Jebaraj B.M.C., Demerdash Y., Öztürk S., Lichter P., Stilgenbauer S., Seiffert M. (2018). PI3Kδ inhibition modulates regulatory and effector T-cell differentiation and function in chronic lymphocytic leukemia. Leukemia.

[196] Petersen C.T., Hassan M., Morris A.B., Jeffery J., Lee K., Jagirdar N., Staton A.D., Raikar S.S., Spencer H.T., Sulchek T. (2018). Improving T-cell expansion and function for adoptive T-cell therapy using ex vivo treatment with PI3Kδ inhibitors and VIP antagonists. Blood Adv..

[197] Dubovsky J.A., Beckwith K.A., Natarajan G., Woyach J.A., Jaglowski S., Zhong Y., Hessler J.D., Liu T.-M., Chang B.Y., Larkin K.M. (2013). Ibrutinib is an irreversible molecular inhibitor of ITK driving a Th1-selective pressure in T lymphocytes. Blood.

[198] Mamontov P., Eberwine R.A., Perrigoue J., Das A., Friedman J.R., Mora J.R. (2019). A negative role for the interleukin-2-inducible T-cell kinase (ITK) in human Foxp3+ TREG differentiation. PLoS ONE.

[199] Hu J., August A. (2008). Naive and innate memory phenotype CD4+ T cells have different requirements for active Itk for their development. J. Immunol..

[200] Kapnick S.M., Stinchcombe J.C., Griffiths G.M., Schwartzberg P.L. (2017). Inducible T Cell Kinase Regulates the Acquisition of Cytolytic Capacity and Degranulation in CD8(+) CTLs. J. Immunol..

[201] Kondo K., Shaim H., Thompson P.A., Burger J.A., Keating M., Estrov Z., Harris D., Kim E., Ferrajoli A., Daher M. (2018). Ibrutinib modulates the immunosuppressive CLL microenvironment through STAT3-mediated suppression of regulatory B-cell function and inhibition of the PD-1/PD-L1 pathway. Leukemia.

[202] Parry H.M., Mirajkar N., Cutmore N., Zuo J., Long H., Kwok M., Oldrieve C., Hudson C., Stankovic T., Paneesha S. (2019). Long-Term Ibrutinib Therapy Reverses CD8(+) T Cell Exhaustion in B Cell Chronic Lymphocytic Leukaemia. Front. Immunol..

[203] Yin Q., Sivina M., Robins H., Yusko E., Vignali M., O’Brien S., Keating M.J., Ferrajoli A., Estrov Z., Jain N. (2017). Ibrutinib Therapy Increases T Cell Repertoire Diversity in Patients with Chronic Lymphocytic Leukemia. J. Immunol..

[204] Hanna B.S., Yazdanparast H., Demerdash Y., Roessner P.M., Schulz R., Lichter P., Stilgenbauer S., Seiffert M. (2020). Combining ibrutinib and checkpoint blockade improves CD8+ T-cell function and control of chronic lymphocytic leukemia in Em-TCL1 mice. Haematologica.

[205] Ruella M., Kenderian S.S., Shestova O., Fraietta J.A., Qayyum S., Zhang Q., Maus M.V., Liu X., Nunez-Cruz S., Klichinsky M. (2016). The Addition of the BTK Inhibitor Ibrutinib to Anti-CD19 Chimeric Antigen Receptor T Cells (CART19) Improves Responses against Mantle Cell Lymphoma. Clin Cancer Res..

[206] Sagiv-Barfi I., Kohrt H.E., Czerwinski D.K., Ng P.P., Chang B.Y., Levy R. (2015). Therapeutic antitumor immunity by checkpoint blockade is enhanced by ibrutinib, an inhibitor of both BTK and ITK. Proc. Natl. Acad. Sci. USA.

[207] Stephens D.M., Byrd J.C. (2019). How I manage ibrutinib intolerance and complications in patients with chronic lymphocytic leukemia. Blood.

[208] Witzig T.E., Nowakowski G.S., Habermann T.M., Goy A., Hernandez-Ilizaliturri F.J., Chiappella A., Vitolo U., Fowler N., Czuczman M.S. (2015). A comprehensive review of lenalidomide therapy for B-cell non-Hodgkin lymphoma. Ann. Oncol..

[209] Vitolo U., Trněný M., Belada D., Carella A.M., Chua N., Abrisqueta P., Demeter J., Flinn I.W., Hong X., Kim W.S. (2016). Obinutuzumab or Rituximab Plus CHOP in Patients with Previously Untreated Diffuse Large B-Cell Lymphoma: Final Results from an Open-Label, Randomized Phase 3 Study (GOYA). Blood.

[210] Alizadeh D., Wong R.A., Yang X., Wang D., Pecoraro J.R., Kuo C.F., Aguilar B., Qi Y., Ann D.K., Starr R. (2019). IL15 Enhances CAR-T Cell Antitumor Activity by Reducing mTORC1 Activity and Preserving Their Stem Cell Memory Phenotype. Cancer Immunol. Res..

[211] Hoffmann J.M., Schubert M.L., Wang L., Huckelhoven A., Sellner L., Stock S., Schmitt A., Kleist C., Gern U., Loskog A. (2017). Differences in Expansion Potential of Naive Chimeric Antigen Receptor T Cells from Healthy Donors and Untreated Chronic Lymphocytic Leukemia Patients. Front. Immunol..

[212] Stock S., Ubelhart R., Schubert M.L., Fan F., He B., Hoffmann J.M., Wang L., Wang S., Gong W., Neuber B. (2019). Idelalisib for optimized CD19-specific chimeric antigen receptor T cells in chronic lymphocytic leukemia patients. Int. J. Cancer.

[213] Arcangeli S., Falcone L., Camisa B., De Girardi F., Biondi M., Giglio F., Ciceri F., Bonini C., Bondanza A., Casucci M. (2020). Next-Generation Manufacturing Protocols Enriching TSCM CAR T Cells Can Overcome Disease-Specific T Cell Defects in Cancer Patients. Front. Immunol..

[214] Kawalekar O.U., O’Connor R.S., Fraietta J.A., Guo L., McGettigan S.E., Posey A.D., Patel P.R., Guedan S., Scholler J., Keith B. (2016). Distinct Signaling of Coreceptors Regulates Specific Metabolism Pathways and Impacts Memory Development in CAR T Cells. Immunity.

[215] Zhao X., Yang J., Zhang X., Lu X.A., Xiong M., Zhang J., Zhou X., Qi F., He T., Ding Y. (2020). Efficacy and Safety of CD28- or 4-1BB-Based CD19 CAR-T Cells in B Cell Acute Lymphoblastic Leukemia. Mol. Ther. Oncol..

[216] Guedan S., Posey A.D., Shaw C., Wing A., Da T., Patel P.R., McGettigan S.E., Casado-Medrano V., Kawalekar O.U., Uribe-Herranz M. (2018). Enhancing CAR T cell persistence through ICOS and 4-1BB costimulation. JCI Insight.

[217] Geiger R., Rieckmann J.C., Wolf T., Basso C., Feng Y., Fuhrer T., Kogadeeva M., Picotti P., Meissner F., Mann M. (2016). L-Arginine Modulates T Cell Metabolism and Enhances Survival and Anti-tumor Activity. Cell.

[218] Li W., Qiu S., Chen J., Jiang S., Chen W., Jiang J., Wang F., Si W., Shu Y., Wei P. (2020). Chimeric Antigen Receptor Designed to Prevent Ubiquitination and Downregulation Showed Durable Antitumor Efficacy. Immunity.

[219] Lynn R.C., Weber E.W., Sotillo E., Gennert D., Xu P., Good Z., Anbunathan H., Lattin J., Jones R., Tieu V. (2019). c-Jun overexpression in CAR T cells induces exhaustion resistance. Nature.

[220] Cherkassky L., Morello A., Villena-Vargas J., Feng Y., Dimitrov D.S., Jones D.R., Sadelain M., Adusumilli P.S. (2016). Human CAR T cells with cell-intrinsic PD-1 checkpoint blockade resist tumor-mediated inhibition. J. Clin. Investig..

