# Correction: van Bruggen et al. Overcoming the Hurdles of Autologous T-Cell-Based Therapies in B-Cell Non-Hodgkin Lymphoma. *Cancers* 2020, *12*, 3837

**DOI:** 10.3390/cancers13194738

**Published:** 2021-09-22

**Authors:** Jaco A. C. van Bruggen, Anne W. J. Martens, Sanne H. Tonino, Arnon P. Kater

**Affiliations:** 1 Department of Hematology, Amsterdam UMC, University of Amsterdam, 1105 AZ Amsterdam, The Netherlands; j.a.vanbruggen@amsterdamumc.nl (J.A.C.v.B.); a.w.martens@amsterdamumc.nl (A.W.J.M.); s.h.tonino@amsterdamumc.nl (S.H.T.); 2 Department of Experimental Immunology, Amsterdam UMC, University of Amsterdam, 1105 AZ Amsterdam, The Netherlands; 3 Cancer Center Amsterdam, 1105 AZ Amsterdam, The Netherlands; 4 Amsterdam Infection & Immunity Institute, 1105 AZ Amsterdam, The Netherlands; 5 Lymphoma and Myeloma Center Amsterdam, LYMMCARE, 1105 AZ Amsterdam, The Netherlands

In the original article [1], reference [46] was not cited correctly in Table 1. The citation has now been inserted in Section 2.1. “Immune Checkpoint Blockade”, Table 1: the original citation “Zinzani et al., 2019” should read “Zinzani et al., 2019 [46]”.

In the original article, reference [84] was not cited correctly in Table 3. The citation has now been inserted in Section 2.3. “Chimeric Antigen Receptor Therapy”, Table 3: the original citation “Fraietta et al., 2016 [78]” should read: “Fraietta et al., 2016 [84]”.

The reference citation numbers from Section 3.2. “T-Cell Skewing”, paragraph two, line three onwards were incorrectly written as a result of four missing references in the reference section. They have been corrected after the references were reinserted with the following numbers: [103], [117], [153] and [192]:

103. Tinhofer, I.; Weiss, L.; Gassner, F.; Rubenzer, G.; Holler, C.; Greil, R. Difference in the relative distribution of CD4+ T-cell subsets in B-CLL with mutated and unmutated immunoglobulin (Ig) VH genes: implication for the course of disease. *J. Immunother.* **2009**, *32*, 302–309, doi:10.1097/CJI.0b013e318197b5e4.
117. Song, D.G.; Ye, Q.; Carpenito, C.; Poussin, M.; Wang, L.P.; Ji, C.; Figini, M.; June, C.H.; Coukos, G.; Powell, D.J., Jr. In vivo persistence, tumor localization, and antitumor activity of CAR-engineered T cells is enhanced by costimulatory signaling through CD137 (4-1BB). *Cancer Res*. **2011**, *71*, 4617–4627, doi:10.1158/0008-5472.CAN-11-0422.
153. Ramsay, A.G.; Johnson, A.J.; Lee, A.M.; Gorgun, G.; Le Dieu, R.; Blum, W.; Byrd, J.C.; Gribben, J.G. Chronic lymphocytic leukemia T cells show impaired immunological synapse formation that can be reversed with an immunomodulating drug. *J. Clin. Investig.* **2008**, *118*, 2427–2437, doi:10.1172/JCI35017.
192. Ramsay, A.G.; Evans, R.; Kiaii, S.; Svensson, L.; Hogg, N.; Gribben, J.G. Chronic lymphocytic leukemia cells induce defective LFA-1-directed T-cell motility by altering Rho GTPase signaling that is reversible with lenalidomide. *Blood* **2013**, *121*, 2704–2714, doi:10.1182/blood-2012-08-448332.

The authors apologize for any inconvenience caused and state that the scientific conclusions are unaffected. The original article has been updated.
